# Gut microbiome and metabolome in a non-human primate model of chronic excessive alcohol drinking

**DOI:** 10.1038/s41398-021-01728-6

**Published:** 2021-12-01

**Authors:** Daria Piacentino, Silvia Grant-Beurmann, Carlotta Vizioli, Xiaobai Li, Catherine F. Moore, Victor Ruiz-Rodado, Mary R. Lee, Paule V. Joseph, Claire M. Fraser, Elise M. Weerts, Lorenzo Leggio

**Affiliations:** 1 Clinical Psychoneuroendocrinology and Neuropsychopharmacology Section, Translational Addiction Medicine Branch, National Institute on Drug Abuse Intramural Research Program and National Institute on Alcohol Abuse and Alcoholism Division of Intramural Clinical and Biological Research, National Institutes of Health, 251 Bayview Blvd, Baltimore, MD 21224 USA; 2 Center on Compulsive Behaviors, National Institutes of Health, 10 Center Dr, Bethesda, MD 20892 USA; 3 Institute for Genome Sciences, Department of Medicine, University of Maryland School of Medicine, Baltimore, MD USA; 4 Sensory Science and Metabolism Unit, National Institute on Alcohol Abuse and Alcoholism Division of Intramural Clinical and Biological Research and National Institute of Nursing Research Division of Intramural Research, 10 Center Dr, Bethesda, MD 20892 USA; 5 Biostatistics and Clinical Epidemiology Services, National Institutes of Health, Bethesda, MD USA; 6 Division of Behavioral Biology, Department of Psychiatry and Behavioral Sciences, Johns Hopkins University School of Medicine, Nathan Shock Drive, Baltimore, MD 21224 USA; 7 Neuro-Oncology Branch, National Cancer Institute, National Institutes of Health, 10 Center Dr, Bethesda, MD 20892 USA; 8 Medication Development Program, National Institute on Drug Abuse Intramural Research Program, National Institutes of Health, 251 Bayview Blvd, Baltimore, MD 21224 USA; 9 Center for Alcohol and Addiction Studies, Department of Behavioral and Social Sciences, Brown University School of Public Health, 121 South Main Street, Providence, RI USA; 10 Division of Addiction Medicine, Department of Medicine, Johns Hopkins University School of Medicine, 733 N Broadway, Baltimore, MD 21205 USA; 11 Department of Neuroscience, Georgetown University Medical Center, 3970 Reservoir Rd NW, Washington, DC 20007 USA

**Keywords:** Addiction, Neuroscience

## Abstract

A relationship between the gut microbiome and alcohol use disorder has been suggested. Excessive alcohol use produces changes in the fecal microbiome and metabolome in both rodents and humans. Yet, these changes can be observed only in a subgroup of the studied populations, and reversal does not always occur after abstinence. We aimed to analyze fecal microbial composition and function in a translationally relevant baboon model of chronic heavy drinking that also meets binge criteria (drinking too much, too fast, and too often), i.e., alcohol ~1 g/kg and blood alcohol levels (BALs) ≥ 0.08 g/dL in a 2-hour period, daily, for years. We compared three groups of male baboons (*Papio anubis*): L = Long-term alcohol drinking group (12.1 years); S = Short-term alcohol drinking group (2.7 years); and C = Control group, drinking a non-alcoholic reinforcer (Tang®) (8.2 years). Fecal collection took place during 3 days of Drinking (D), followed by a short period (3 days) of Abstinence (A). Fecal microbial alpha- and beta-diversity were significantly lower in L vs. S and C (*p*’s < 0.05). Members of the commensal families Lachnospiraceae and Prevotellaceae showed a relative decrease, whereas the opportunistic pathogen *Streptococcus* genus showed a relative increase in L vs. S and C (*p*’s < 0.05). Microbiota-related metabolites of aromatic amino acids, tricarboxylic acid cycle, and pentose increased in L vs. S and C (FDR-corrected *p* < 0.01), with the latter two suggesting high energy metabolism and enhanced glycolysis in the gut lumen in response to alcohol. Consistent with the long-term alcohol exposure, mucosal damage and oxidative stress markers (N-acetylated amino acids, 2-hydroxybutyrate, and metabolites of the methionine cycle) increased in L vs. S and C (FDR-corrected *p* < 0.01). Overall, S showed few differences vs. C, possibly due to the long-term, chronic alcohol exposure needed to alter the normal gut microbiota. In the three groups, the fecal microbiome barely differed between conditions D and A, whereas the metabolome shifted in the transition from condition D to A. In conclusion, changes in the fecal microbiome and metabolome occur after significant long-term excessive drinking and are only partially affected by acute forced abstinence from alcohol. These results provide novel information on the relationship between the fecal microbiome and metabolome in a controlled experimental setting and using a unique non-human primate model of chronic excessive alcohol drinking.

## Introduction

Excessive alcohol use is a serious public health problem that accounts for one in 10 deaths annually, among adults aged 20–64 years, in the United States [1]. Excessive alcohol use includes binge drinking and heavy drinking. Binge drinking (drinking too much, too fast) is a pattern of drinking that brings blood alcohol levels (BALs) to 0.08 g/dL, or above, in about 2 hours and corresponds to 0.8–1 g/kg of body weight or > 4 drinks/occasion for men and > 3 drinks/occasion for women [2]. Heavy drinking (drinking too much, too often) is defined as drinking > 14 drinks/week for men and > 7 drinks/week for women [3]. Binge and heavy drinking increase the risk of alcohol use disorder (AUD).

The effects of alcohol on the fecal microbiome and metabolome have been studied in both animal models and individuals with AUD. Alcohol-induced changes in gut microbial composition (dysbiosis) and metabolic function may contribute, together with direct alcohol toxicity, to gut barrier dysfunction, bacterial translocation, inflammation, and, ultimately, the development of alcohol-associated liver disease (ALD) [4–7]. The gut microbiota harbors a community of bacteria that, for the most part, belong to the phyla Bacteroidetes, Firmicutes, Actinobacteria, and Proteobacteria [8]. These bacteria take part in the generation of metabolites derived from dietary or endogenous sources of carbohydrates, lipids, and proteins [9].

The first studies to demonstrate gut dysbiosis resulting from alcohol use were conducted in rats [10] and mice [11, 12]. Since then, a number of rodent studies testing different models of alcohol administration have followed; they report a significant loss in the overall diversity of the fecal microbial community [13, 14], as well as changes in the relative abundance of microbial taxa, such as Bacteroidetes and Firmicutes [13–20]. Consistent with these studies, preliminary work from our group in male Wistar rats shows that alcohol binge-like exposure induced both a decrease in fecal microbial alpha- and beta-diversity and changes in the proportion of *Bacteroidales* order and *Paraprevotella* and *Turicibacter* genera [21]. These rats were tested in an operant paradigm with a sweetened 10% w/v alcohol solution, which has been demonstrated to generate alcohol consumption to the point of binge levels [22]. A minority of rodent studies used fecal metabolomics to investigate the effects of chronic alcohol administration on metabolism by the gut microbiota; they show varying degrees of changes in bile acids (BAs), amino acids, fatty acids, and phenols [15, 20, 23, 24]. It is unclear how accurately the findings from rodents translate to humans, due to the evolutionary distance between the two species [25] and the dissimilar alcohol metabolism rate [26] and gut microbial composition [27].

Human studies point to an association between gut dysbiosis and chronic alcohol consumption in alcohol-dependent individuals, both with and without ALD [4–7, 28–36]. The individuals with greater gut dysbiosis show increased intestinal permeability and transfer of bacterial products into the bloodstream [4, 5, 7, 36], which induce the release of pro‐inflammatory mediators such as reactive oxygen species, chemokines, and cytokines [37]; they are also more likely to have liver damage [6, 28, 33]. Yet, gut dysbiosis can be observed only in a subset of individuals with chronic excessive alcohol use [4, 5], reversal of dysbiosis does not always occur after abstaining from alcohol [4, 5, 7], and the presence in some individuals of advanced ALD and the related additional shifts in fecal microbial taxa represent a confounder [6, 30, 33].

Studies on alcohol-induced alterations in the fecal metabolome are not as numerous as those on the fecal microbiome. Chronic alcohol consumption has been linked to changes in amino acids, BAs, lipids, neurotransmitters, and markers of oxidative stress, likely reflecting alterations in gut bacteria [32, 38, 39]. Most of these studies, however, do not account for lifestyle and environmental factors that affect the fecal microbiome and metabolome, such as the type and amount of alcohol consumed, diet, smoking, illicit drug use, comorbidities, and medication use [40].

In this respect, non-human primate (NHP) models are exceptionally valuable for translational research, providing a degree of experimental control that is not possible in humans [41–43]. Up to now, only two studies have investigated the effects of excessive alcohol use on the gut microbiome in NHPs, specifically male macaques (*Macaca mulatta*) [44, 45], and only one of them performed fecal metabolomics [45]. One study reported alcohol-induced loss of fecal microbial alpha-diversity and differences in glycolysis and fatty acid metabolism [45], similar to previous findings in rodents and humans, whereas the second study did not identify differences in taxonomic diversity of intestinal mucosal biopsies following chronic alcohol self-administration [44]. This discrepancy may be attributed to differences between mucosal and luminal microbial communities, as well as quantity and/or duration of alcohol consumption and operating procedures.

The present study provides a unique opportunity to expand on our prior work in rats [21] and investigate whether chronic heavy drinking that also meets binge criteria is associated with specific shifts of the fecal microbiome in NHPs, specifically male baboons (*Papio anubis*). Furthermore, we used this model to assess whether chronic excessive alcohol drinking is associated with significant changes in fecal metabolites related to gut dysbiosis. Animals in this study self-administered alcohol under a chained schedule of reinforcement (CSR) operant procedure [46–48]. The CSR procedure models both binge (too much, too fast) and heavy (too much, too often) drinking patterns as defined by the NIAAA, by intake in a 2-hour period resulting in BALs ≥ 0.08 g/dL and consumption of ~1 g/kg/day of alcohol, 7 days/week [2, 3]. The alcohol drinking baboons were compared to a control group that self-administered a preferred non-alcoholic beverage (Tang®) under the same CSR procedure. This NHP model has several advantages: (1) baboons are closely related to humans in phylogeny, anatomy, physiology, neurochemistry, and behavior [42]; (2) they allow long-term voluntary alcohol consumption (years) in a controlled environment due to their long lifespan (~45 years) [42]; (3) they metabolize alcohol similarly to humans and their alcohol intake (g/kg) produces BALs that mirror those in humans [42]; and (4) their gut microbiota is comparable to that of humans and it has been suggested that captivity humanizes the NHP microbiota [43]. Fecal microbiome and metabolome analysis in baboons can provide important cross-species validation to bridge the translational gap in alcohol research between rodents and humans.

## Materials and methods

### Animals

Animals (*N* = 16) in this study were adult male baboons (*Papio anubis*), also known as olive baboons. Fourteen baboons had extensive histories of self-administration under the CSR procedure. Based on history of exposure, baboons were categorized into one of three groups (Table 1): L = Long-term alcohol drinking group, self-administering alcohol daily for a median of 12.1 years (*N* = 4); S = Short-term alcohol drinking group, self-administering alcohol daily for a median of 2.7 years (*N* = 5); and C = Control group, self-administering Tang, an orange-flavored, non-alcoholic beverage, daily for a median of 8.2 years (*N* = 5). The 14 baboons underwent fecal collection during three consecutive days of alcohol/Tang Drinking (condition D, days 1–3), followed by the first three days of alcohol/Tang Abstinence (condition A, days 4–6) (Table 1). Two additional baboons (individual animal identifiers: HB and SHA) were used only for blood sampling for BAL to provide an additional control; they did not self-administer alcohol or Tang under the CSR, were drug-free, and were maintained in the same housing and feeding conditions as the other 14 baboons.

**Table 1:** Baboons and sampling time points for each group and condition.

Individual animal identifiers	Group	Condition	Sampling time point (Total *N* of samples)		
			Time point 1	Time point 2	Time point 3
DK	Long-term alcohol drinking group (L)	Drinking	4 (1 per animal)	4 (1 per animal)	4 (1 per animal)
RAF					
RS		Abstinence	4 (1 per animal)	4 (1 per animal)	4 (1 per animal)
WI					
CY	Short-term alcohol drinking group (S)	Drinking Abstinence	5 (1 per animal) 5 (1 per animal)	5 (1 per animal) 5 (1 per animal)	5 (1 per animal) 5 (1 per animal)
GB					
HA					
HS					
LN					
BA	Control group (C)	Drinking Abstinence	5 (1 per animal) 5 (1 per animal)	5 (1 per animal) 5 (1 per animal)	5 (1 per animal) 5 (1 per animal)
DN					
KI					
NO					
PR					

Groups included the long-term alcohol drinking group, the short-term alcohol drinking group, and the Tang® control group. The baboons in each group were as follows: four in group L (DK, RAF, RS, WI), five in group S (CY, GB, HA, HS, LN), and five in group C (BA, DN, KI, NO, PR). Sampling conditions included three consecutive days of drinking, followed by three consecutive days of abstinence.

The baboons were singly housed, in modified primate cages, in the vivarium at the Johns Hopkins School of Medicine, Baltimore, MD, USA. The housing room was maintained under a 12-hour light/dark cycle, with lights on from 6:00 AM to 6:00 PM daily. The cages also served as the experimental chambers and were fitted with operant intelligence panels, as described in detail previously [46]. Briefly, the solutions for oral consumption were available for self-administration from a “drinkometer” with a protruding drink spout (Kandota Instruments, Sauk Center, MN, USA) connected to a calibrated 1000-mL bottle, and only during the final component of the CSR, as detailed below. A separate panel on the back wall contained three colored cue lights (red, yellow, and blue). A speaker was mounted above the cages for presentation of auditory stimuli (tones). Experimental events were controlled remotely using Med Associates (East Fairfield, VT, USA) software and hardware interfaced with a personal computer. Cages were positioned to allow full visual, auditory, and olfactory contact with the other singly housed baboons in the room. Diet was similar among animals. Baboons were fed daily with primate chow (50–73 kcals/kg), two pieces of fresh fruit or vegetable, and a children’s chewable multivitamin. This diet enables baboons to maintain a relatively stable weight, while slowly gaining weight as they age [49]. Tap water from a drinking spout located on the front of the cage was available ad libitum, except during experimental sessions. The facilities were maintained in accordance with USDA and AAALAC standards and followed the Guide for the Care and Use of Laboratory Animals (National Research Council, 2011). The protocol was approved by the Johns Hopkins University Animal Care and Use Committee.

### Alcohol and tang self-administration under the chained schedule of reinforcement (CSR)

The CSR procedures used in the current study were identical for the alcohol and Tang groups, as reported in the Supplementary Methods and described in detail previously [47]. The CSR sessions ran daily (7 days/week) and both alcohol and Tang groups sessions began at the same time each day (e.g., 8:30 AM). Immediately following each session, alcohol intake (mL) was recorded. The CSR consisted of three separate sequential components, each of which was associated with distinct stimuli and behavioral contingencies. Fulfilling the schedule requirement(s) in each component was necessary to proceed to the next, with access to alcohol or Tang for self-administration for 2 h in the final component of the CSR. Indeed, relatively stable levels of alcohol intake can be obtained and maintained over consecutive days of alcohol availability when access is limited to 2 h/day [50]. During these 2 h, contact with the spout operated a solenoid valve that delivered the solutions for 5 sec (i.e., 1 drink ~30 mL). All solutions were mixed using reverse osmosis (RO) purified drinking water. Ethyl alcohol (190 Proof, Pharmco-AAPER, Brookville, CT, USA) was diluted with RO water to 4% w/v alcohol. Orange-flavored, Tang powder (Kraft Foods®) was dissolved in RO water following package instructions and then diluted from full strength (100%) to concentrations of 25% and 50% for two (BA, PR) and three baboons (DN, KI, NO) of group C, respectively. Previous CSR studies determined breaking points for 2–8% w/v alcohol and 25–100% Tang solutions under progressive ratio procedures to determine relative reinforcement [47, 48]. The Tang concentrations used in the current study were those that produced comparable breaking points to 4% alcohol (i.e., functionally equivalent reinforcer). In condition A, alcohol and Tang availability was withheld from the self-administering animals. By limiting alcohol access to 2 h/day, baboons typically only show mild symptoms of alcohol withdrawal (e.g., irritability, inactivity, and reduced food intake). This restriction is intentional to avoid complications due to withdrawal [51] and to model one of the most common patterns of excessive alcohol use.

On days when blood collection was scheduled to measure BAL, baboons were anesthetized with ketamine and 5-mL blood samples were collected from a saphenous vein. Samples were centrifuged at 3200 rpm for 8–12 min and then the plasma drawn off, transferred to two separate airtight polypropylene tubes, and frozen until analysis. Similar to our prior studies [47], double determinations of BAL were completed using a rapid high performance plasma alcohol analysis using alcohol oxidase with an Analox GM7 MicroStat Analyzer (Analox Instruments USA, Lenenberg, MA, USA), and a GMRD-113 Alcohol reagent kit, with GMRD-110 internal ethanol standards of 300 mg/dL (detects ranges from 0 to 350 mg/dL).

On the days the samples were collected, all the baboons in groups S and L drank physiologically relevant amounts of alcohol (range 0.63–1.15 g/kg/day); the resulting BALs exceeded 0.08 g/dL (range: 0.09–0.24 g/dL). BALs in the baboons consuming Tang in group C ranged 0.003–0.015 g/dL and were comparable to those (range: 0.013–0.024 g/dL) of the two baboons (HB, SHA) without any access to alcohol or Tang. These BALs may be achieved through endogenous alcohol production, deriving primarily from the gut microbial fermentation of carbohydrates [52]. Endogenous alcohol is normally present in minimal quantities in the blood of abstaining individuals, with BALs ranging 0–0.07 g/dL [53].

Blood collection also served to measure the levels of cortisol and the following cytokines: interleukin (IL)-1β, IL-4, IL-5, IL-6, IL-12p70, IL-15, IL-16, interferon-gamma (IFN-γ), tumor necrosis factor-alpha (TNF-α), and granulocyte-macrophage colony-stimulating factor (GM-CSF), as described in the Supplementary Methods.

### Fecal sample collection

One fecal sample per animal was collected at each sampling time point, for a total of six fecal samples per animal (three per condition) (Table 1).

Collection of feces via a sterile collector pan occurred in the afternoon after the baboons ate. Pans were checked every 15 min to ensure collection of fresh feces. The collected feces were visually inspected to exclude contamination with urine and were removed from the pan using a spatula; a sample was taken from the interior of the feces using another spatula to capture both the aerobic and anaerobic microbial communities. The fecal sample was divided into two aliquots: one (5 mL) for microbiome analysis, which was transferred into a 15 mL Fisherbrand graduated conical-bottom tube (ThermoFisher Scientific, Waltham, MA, USA) containing Invitrogen RNA*later* Stabilization Solution (~6 mL; ThermoFisher Scientific, Waltham, MA, USA); the other one (5 mL) for metabolome analysis, which was transferred into a 15 mL Fisherbrand graduated conical-bottom tube (ThermoFisher Scientific, Waltham, MA, USA). All samples were immediately stored at −80 °C until processing. No animals or samples were excluded from the analyses. Consistent with the study design, randomization or blinding procedures did not apply.

### Fecal microbiome analysis

The fecal samples containing RNA*later* were sent to the Institute for Genome Sciences, University of Maryland School of Medicine, Baltimore, MD, USA, for DNA extraction and next generation 16S rRNA gene sequence analysis. DNA was extracted from each fecal specimen. Samples were thawed at 4 °C and, in aliquots of 0.15 g per tube, resuspended in 1 mL of 1 x phosphate-buffered saline. Cell lysis was initiated with two enzymatic incubations: (1) using 5 µL of lysozyme (10 mg/mL; Amresco, Solon, OH, USA), 13 µL of mutanolysin (11.7 U/µL; Sigma–Aldrich, St. Louis, MO, USA), and 3 µL of lysostaphin (4.5 U/µL; Sigma–Aldrich, St. Louis, MO, USA) for an incubation of 30 min at 37 °C, and (2) using 10 µL of proteinase K (20 mg/mL; Research Products International, Mt. Prospect, IL, USA), 50 µL of 10% SDS, and 2 µL of RNase (10 mg/mL) for an incubation of 45 min at 56 °C. After the enzyme treatments, cells were disrupted by bead beating in tubes with lysing matrix B (0.1-mm silica spheres; MP Biomedicals, Solon, OH, USA), at 6 m/sec at room temperature in a FastPrep-24 (MP Biomedicals, Santa Ana, CA, USA). The resulting crude lysate was processed using the Zymo Research fecal DNA miniprep kit (Zymo, Irvine, CA, USA), according to the manufacturer’s recommendations. The samples were eluted with 100 µL of ultrapure sterile water into separate tubes. DNA concentrations in the samples were determined with the Bioanalyzer 2100 DNA 1000 chip (Agilent, Santa Clara, CA, USA).

Hypervariable regions V3 and V4 of the bacterial 16S rRNA gene were amplified with primers 319F and 806R [54]. The libraries were sequenced on 250 paired-end Illumina HiSeq runs. The raw sequence data were demultiplexed with the software Trimmomatic. For assembly of the reads, FLASH software was used, which utilizes the output by Trimmomatic to merge paired-end reads to make contigs. The minimum overlap between two reads to provide a confident overlap was set to 30 base pairs. A QIIME-dependent script was used to assign the preprocessed reads to the samples. The index and barcode sequences were removed and, from that point, a mismatch of only two base pairs was allowed. The UCHIME2 algorithm [55] implemented in USEARCH was used to perform a reference-based chimera removal using the GreenGenes dataset as a reference. Sequences were clustered as operational taxonomic units (OTUs) based on a 97% cutoff with USEARCH. Taxonomic ranks were assigned to each sequence with the Ribosomal Database Project Naive Bayesian Classifier version 2.2, using the GreenGenes database of 16S rRNA gene sequences (August 2013) and a confidence value cutoff of 0.97. Sequence reads from the 16S rRNA gene profiling are available at Sequence Read Archive under accession number SRR8514479.

### Fecal metabolome analysis

The fecal samples were sent to Metabolon Inc., Morrisville, NC, USA, for metabolomics analysis by using untargeted ultra-performance liquid chromatography-tandem mass spectrometry (UPLC/MS/MS, Waters ACQUITY, Milford, MA, USA), as reported in the Supplementary Methods and described in detail previously [56–58]. Briefly, fecal samples were prepared using the automated MicroLab STAR system (Hamilton Company, Franklin, MA, USA) and extracted at a constant per-mass basis. Proteins and other macromolecules were removed using methanol precipitation (Glen Mills GenoGrinder 2000), followed by centrifugation. Samples were run over the course of a single day, using four methods, against three controls (a pooled sample, extracted water to serve as process blank, and a cocktail of quality control standards to serve as technical replicates). The four methods used were reverse phase (RP)-UPLC/MS/MS with electrospray ionization (ESI), in both positive (optimized for hydrophilic and hydrophobic compounds, respectively) and negative modes, and hydrophilic interaction chromatography (HILIC)-UPLC/MS/MS-ESI in negative ion mode. The raw UPLC/MS/MS data were integrated into ion peaks organized by mass (including adducts, isotopes, multimers, and in-source fragments), retention time/index, and peak area. Metabolites were identified by comparison of individual spectra to a standard reference library, and area-under-the-curve analysis was performed for peak quantification. Metabolite values were median-scaled, and the resulting dataset, which was used for univariate and multivariate analyses, comprised 534 metabolites measured in the 84 fecal samples from the 14 baboons, all of which had known chemical identities.

### Computing and statistics

#### Subject characteristics

We compared the main characteristics of the three groups of baboons, i.e., age, weight, and levels of liver enzymes, cytokines, and cortisol, using the non-parametric Kruskal–Wallis test, due to the non-normal distribution of the data (Shapiro–Wilk test = 0.945–0.998, *p*’s = 0.236–0.375). A *p* < 0.05 (two-tailed) was considered statistically significant. The analyses were performed with R version 3.6.3.

#### Microbiome analysis

**Fig. 1:**
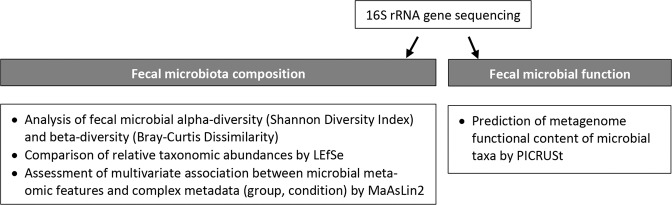
Fecal microbiome data analysis workflow. After DNA extraction and next generation 16S rRNA gene sequence analysis, fecal microbiota composition and function were assessed. The study of fecal microbiota composition included alpha- and beta-diversity measurements, the comparison of relative taxonomic abundances using linear discriminant analysis effect size (LEfSe), and the assessment of the relationship between clinical metadata (groups of baboons; drinking and abstinence conditions) and microbial community abundance using multivariate microbial association by linear models (MaAsLin2). The study of fecal microbiota function was based on the analysis of genetic functional potential using phylogenetic investigation of communities by reconstruction of unobserved states (PICRUSt) based on the Kyoto Encyclopedia of Genes and Genomes (KEGG) orthologs.

For fecal microbiota profiling, sample counts were rarefied and trimmed for the consequently absent OTUs with the phyloseq R package [59] based on genus estimates < 1%. Statistical analysis was conducted on the rarefied and trimmed feature abundance matrices. The workflow of fecal microbiome analysis is illustrated in Fig. 1.

Shannon Diversity Index was used to measure alpha-diversity (within-sample diversity). Family and genus-level analyses were performed using the phyloseq package. We carried out two-way repeated measures ANOVA to assess the main effects of group (L, S, and C), condition (D and A), and group x condition (GxC) interaction, with the six sampling time points as a repeated measure. *Post‐hoc* Tukey’s honestly significant difference test was run for pairwise comparisons. A *p* < 0.05 (two-tailed) was considered statistically significant. A principal coordinates analysis (PCoA) of Bray-Curtis distances (Bray-Curtis Dissimilarity Matrix) was performed to measure beta-diversity (between-sample diversity). A permutational ANOVA (PERMANOVA) tested whether the microbial communities sequenced had different centroids based on exposure or genotype. Significance of the results was confirmed with a test of heterogeneity (to ensure homogenous dispersion). Microbiota changes focusing on relative abundances of bacterial taxa were compared within and between samples using linear discriminant analysis (LDA) effect size (LEfSe) [60]. The Galaxy implementation of LEfSe (http://huttenhower.org/galaxy) with default options was used. Differences were analyzed using Kruskal–Wallis test followed by post-hoc pairwise Wilcoxon test; a *p* < 0.05 (two-tailed) was set for statistical significance, and a size-effect threshold of 2.0 for the logarithmic LDA score was applied for discriminative microbial features. To identify significant associations between clinical metadata (groups L, S, and C; conditions D and A) and microbial community abundance, we applied the multivariate microbial association by linear models (MaAsLin2) statistical framework by using the R package Maaslin2 [61]. MaAsLin2 performs boosted, additive general linear models between one group of data (in our case metadata/the predictors) and another group (in our case microbial abundance/the response). Boosting of metadata and selection of a model occurs per OTU. The data that are selected by boosting are then used in a general linear model, with metadata as predictors and OTU arcsin-square root transformed abundance as the response. We conducted a phylogenetic investigation of communities by reconstruction of unobserved states (PICRUSt) to predict metagenome functional content from the 16S rRNA gene-based microbial compositions using the Kyoto Encyclopedia of Genes and Genomes (KEGG) catalog [62]. By using known genomes, PICRUSt infers abundance of genes based on the abundance of OTUs.

#### Metabolomics analysis

**Fig. 2:**
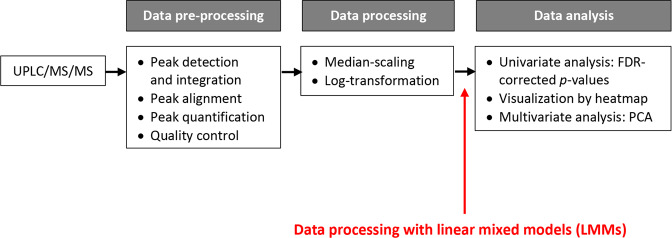
Fecal metabolomics data analysis workflow. The acquisition of raw data from the ultra-performance liquid chromatography-tandem mass spectrometry (UPLC/MS/MS) platform was followed by two steps to generate “clean” data to be used as the input for the statistical analysis (FDR-corrected *p* values, principal component analysis, [PCA]) and biological interpretation: data pre-processing, which included peak alignment and quantification, and data processing, which consisted of scaling and normalization by log-transformation. In our study, the linear mixed models (LMM) method (in red) is proposed as an additional step performed after the conventional data processing tasks.

Median-scaled metabolite values derived from the UPLC/MS/MS analysis of fecal extracts were normalized by log transformation. The workflow of fecal metabolome analysis is illustrated in Fig. 2.

Metabolic profiling is affected by both noise from technical analysis and undesired signals from subject-specific variations, but conventional data processing methods can remove only the former. In our study, linear mixed-effects models (LMMs) (in red in Fig. 2) served as an additional step, performed after the conventional data processing tasks, to account for sources of subject differences and further improve the quality of the dataset [63]. Each of the 534 metabolites was analyzed by LMMs to account for the correlation of the repeated measures within a baboon. The models included group (L, S, and C), condition (D and A), and group x condition (GxC) interaction as the fixed effects. The exchangeable working correlation matrix was assumed, and the robust sandwich estimates were obtained through the “empirical” option in the SAS procedure PROC MIXED (SAS Institute Inc. Cary, NC). All *p* values (two-tailed) for the GxC interaction effect were false discovery rate (FDR)-corrected with an alpha level of 0.01 by using the R package qvalue [64]. A heatmap of the metabolites with a significant GxC interaction in the LMMs was created with the R package pheatmap. A principal components analysis (PCA) was performed to explore how these metabolites were related with the groups and conditions based on the LMM output via the package lmm2met in R.

All R analyses were conducted in R version 3.4.1 or later.

## Results

### Demographic and laboratory characteristics of the sample

The three groups of baboons did not differ significantly in age and weight, and presented with normal liver function, as determined by the veterinarians’ clinical evaluation and standard liver tests (Table 2). Except for a nominal difference in IL-4 levels, which were marginally lower in the L and S groups than in the C group, there were no significant differences in the other inflammatory cytokines or cortisol among the three groups (Supplementary Table 1).

**Table 2:** Baboons’ main demographic and biochemical characteristics.

Variable	Long-term alcohol drinking (*N* = 4)	Short-term alcohol drinking (*N* = 5)	Control (*N* = 5)	*p* value
Age in years, median (IQR)	17.5 (1.7)	15.0 (3.5)	15.0 (2.5)	0.235
Weight in kg, median (IQR)	27.6 (9.1)	28.4 (6.6)	25.9 (10.1)	0.591
ALT in U/L, median (IQR)	23.0 (7.5)	20.5 (49.0)	25.0 (10.7)	0.644
AST in U/L, median (IQR)	24.0 (8.7)	22.5 (19.7)	19.5 (5.5)	0.305
ALP in U/L, median (IQR)	115.0 (14.7)	91.0 (97.3)	99.5 (53.5)	0.332

Medians and interquartile ranges (IQR) are shown for each variable. Kruskal–Wallis test was used to compare the median values of each variable among the three groups of baboons, i.e., the long-term alcohol drinking group, the short-term alcohol drinking group, and the control group. The normal range of alanine transferase (ALT) is 23–69 U/L, the normal range of aspartate transferase (AST) is 19–62 U/L, the normal range of alkaline phosphatase (ALP) is 0–555 U/L.

### Effects of chronic excessive alcohol drinking on fecal microbiota

**Fig. 3:**
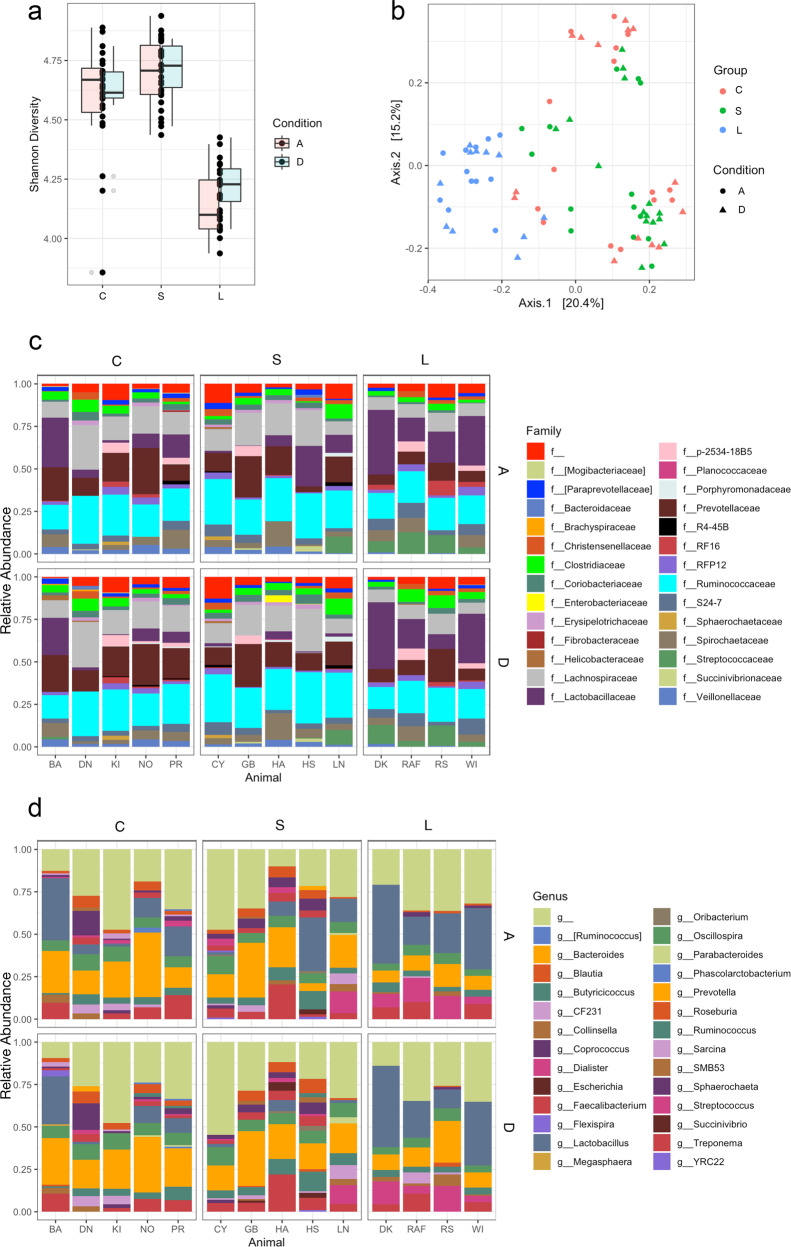
Effects of chronic excessive alcohol drinking on fecal microbiota diversity and composition. **a** Box plots comparing fecal microbial alpha-diversity (Shannon Diversity Index) among the long-term alcohol drinking group (L), the short-term alcohol drinking group (S), and the control group (C). Box plots in pink indicate the drinking condition (D), box plots in turquoise indicate the abstinence condition (A). Each black dot corresponds to a sample. **b** Principal coordinates analysis (PCoA) plot comparing fecal microbial beta-diversity (Bray-Curtis Dissimilarity Matrix) among the groups L (in blue), S (in green), and C (in red). Triangles represent condition D and circles represent condition A. The percent explained dissimilarity is reported in brackets on first (*x* axis) and second (*y* axis) principal coordinate axis. The distance between the triangles and circles, respectively, represents the difference in community composition of the groups of baboons. **c** Family-level fecal microbial composition of group L vs. group S vs. group C (relative abundance > 1%; families with relative abundance < 1% were pooled in the “f” category). Conditions D and A are combined. **d** Genus-level fecal microbial compositions of group L vs. group S vs. group C (relative abundance > 2%; genera with relative abundance < 2% were pooled in the “g” category). Conditions D and A are combined.

A total of 98 million high-quality sequence reads from bacterial 16S rRNA gene V3-V4 amplicons, with a mean ± standard deviation (SD) of 87,895 ± 20,11 reads per sample, were generated (Supplementary Fig. 1). Fecal microbial alpha-diversity, as calculated with the Shannon Diversity Index, showed significant differences among the three groups L, S, and C (F_2,11_ = 24.2, *p* < 0.01), but not between the sampling conditions D and A (F_1,67_ = 0.6, *p* = 0.41). Pairwise comparisons indicated that overall alpha-diversity was significantly lower in the L group compared with the S and C groups (*p* < 0.01 for both comparisons). In contrast, no significant shift was observed when groups S and C were compared (*p* = 0.22) (Fig. 3a). The PCoA using Bray-Curtis distance showed significantly lower fecal microbial beta-diversity in group L and a separation of the latter from the other two groups S and C, accounting for 22.0% of the differences (*p* < 0.01) (Fig. 3b); however, the homogeneity of dispersion test suggested a heterogenous dispersion of the communities (*p* < 0.01), thus the PERMANOVA result may have been influenced by differences in composition within the groups (Supplementary Table 2). Fecal microbiota composition varied across groups and conditions. At the family taxonomic level, Lactobacillaceae and Streptococcaceae were enriched in group L compared with group S. The Prevotellaceae and Lachnospiraceae relative abundance was relatively similar in groups S and C, but decreased in group L. The abundance of Ruminococcaceae slightly increased from group C to group S, followed by a decrease in group L. In group L, RF16 appeared to slightly increase from condition D to condition A (Fig. 3c). Classification down to the genus taxonomic level confirmed the higher proportion of *Lactobacillus* and *Streptococcus* in the L group compared with the S and C groups. *Prevotella* showed a lower relative abundance in the L group compared with the S and C groups. Two genera within the family Lachnospiraceae, *Blautia*, and *Coprococcus*, decreased in group L. An increase of *Lactobacillus* from condition D to condition A in all the three groups of baboons was observed, however this trend was not significant. The reduction in fecal microbial alpha-diversity in group L, as suggested by the Shannon Diversity Index (Fig. 3a), is clearly visible in the microbiota composition graph (Fig. 3d).

**Fig. 4:**
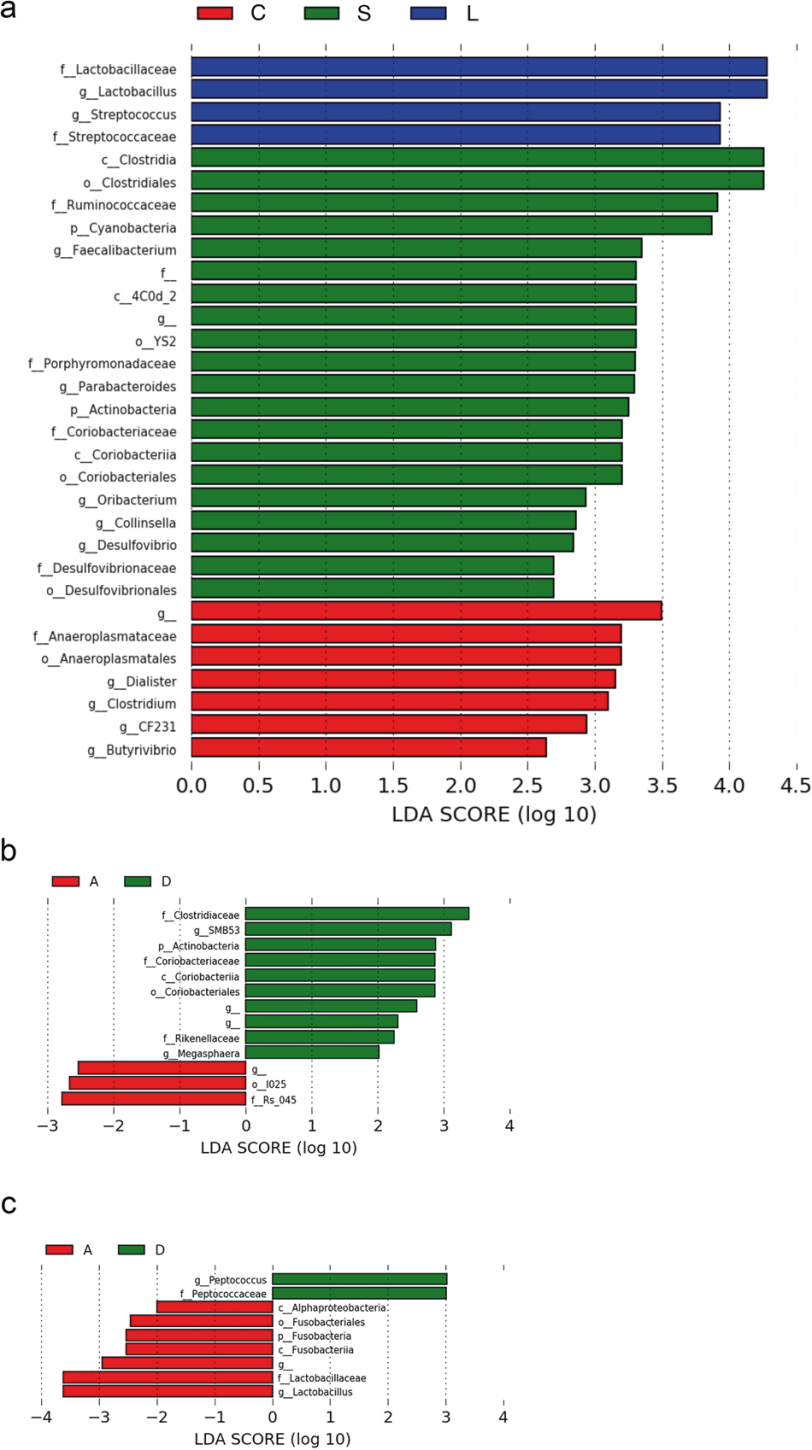
Effects of chronic excessive alcohol drinking on fecal microbiota composition based on linear discriminating analysis (LDA) effect size (LEfSe) results. **a** LEfSe plot at the last known taxon of fecal microbial samples of the long-term alcohol drinking group (L, in blue) vs. the short-term alcohol drinking group (S, in green) vs. the control group (C, in red). The drinking (D) and abstinence (A) conditions are combined. The bars represent the effect size (LDA) for a particular taxon in a certain group of baboons. The length of the bar represents a log10 transformed LDA score. The colors represent the group in which that taxon was found to be more abundant compared to the other groups. f_ indicates that the operational taxonomic unit (OTU) was not able to be classified to a family. g_ indicates that the OTU was not able to be classified to a genus. **b** LEfSe plot at the last known taxon of fecal microbial samples of drinking (D, in green) vs. abstinent (A, in red) baboons in group L. The bars represent the effect size (LDA) for a particular taxon in a certain condition. The length of the bar represents a log10 transformed LDA score. The colors represent the condition in which that taxon was found to be more abundant compared to the other condition. g_ indicates that the OTU was not able to be classified to a genus. **c** LEfSe plot at the last known taxon of fecal microbial samples of drinking (D, in green) vs. abstinent (A, in red) baboons in group S. The bars represent the effect size (LDA) for a particular taxon in a certain condition. The length of the bar represents a log10 transformed LDA score. The colors represent the condition in which that taxon was found to be more abundant compared to the other condition. g_ indicates that the OTU was not able to be classified to a genus.

When comparing the three groups (conditions D and A combined), the LEfSe algorithm identified two genera, *Lactobacillus* and *Streptococcus*, showing a significant increase in group L; as reflected by the LDA score, five genera, *Faecalibacterium*, *Parabacteroides, Oribacterium*, *Collinsella*, and *Desulfovibrio* were consistently enriched in group S; and samples from the baboons in group C exhibited a significantly increased abundance of four genera, *Dialister*, *Clostridium*, *CF231*, and *Butyrivibrio* (Fig. 4a; Table 3). When comparing conditions D and A within each group, LEfSe determined that in group C no taxa exhibited significant differences in relative abundance between the two conditions. When the LEfSe tool was applied to groups L and S, respectively, eight taxa in the first group and two taxa in the second group were significantly more represented during condition D; yet these taxa showed <1% relative abundance in each sample (Fig. 4b, c). MaAsLin2 results aligned with those from LEfSe. When the three groups were compared (conditions D and A combined) (Table 3; Supplementary Table 3), similarly to LEfSe, *Lactobacillus* and *Streptococcus* genera were significantly enriched in the L group compared with the S and C groups. In addition, four genera, *Prevotella*, *Blautia*, *Dialister*, and *Sutterella*, were significantly decreased in the L group compared with the other two groups. In group S, in line with LEfSe, the three genera *Faecalibacterium*, *Parabacteroides*, and *Oribacterium* were significantly increased. An additional genus, *Eubacterium*, was significantly more represented in this group. Three genera, *Butyrivibrio* (similarly to LEfSe), *Dorea*, and *Ruminococcus*, showed a significant increase in group C. When comparing conditions D and A, a significant increase of *Lactobacillus* genus was observed in all the three groups in condition A, in line with LEfSe. A slight decrease of five genera, *Oribacterium*, *Oscillospira*, *Prabacteroides*, *Roseburia*, and *Colinsella*, was also observed in all the three groups in condition A (Supplementary Table 4).

**Table 3:** Changes in fecal microbial taxa based on the results of linear discriminant analysis effect size (LEfSe) and multivariate microbial association by linear models (MaAsLin2).

Phylum	Class	Order	Family	Genus	LEfSe			MaAsLin2		
					D + A			D + A		
					L vs. S	L vs. C	S vs. C	L vs. S	L vs. C	S vs. C
Firmicutes	*Bacilli*	*Lactobacillales*	*Lactobacillaceae*	*Lactobacillus*	↑	↑	NS	↑	↑	NS
Firmicutes	*Bacilli*	*Lactobacillales*	*Streptococcaceae*	*Streptococcus*	↑	↑	NS	↑	↑	NS
Firmicutes	*Clostridia*	*Clostridiales*	*Ruminococcaceae*	*Faecalibacterium*	↓	NS	↑	↓	NS	↑
Bacteroidetes	*Bacteroidia*	*Bacteroidales*	*Porphyromonadaceae*	*Parabacteroides*	↓	NS	↑	↓	NS	↑
Firmicutes	*Clostridia*	*Clostridiales*	*Lachnospiraceae*	*Oribacterium*	↓	NS	↑	↓	NS	↑
Actinobacteria	*Actinobacteria*	*Coriobacteriales*	*Coriobacteriaceae*	*Colinsella*	↓	NS	↑	NS	NS	NS
Proteobacteria	*Deltaproteobacteria*	*Desulfovibrionales*	*Desulfovibrionaceae*	*Desulfovibrio*	↓	NS	↑	NS	NS	NS
Firmicutes	*Negativicutes*	*Selenomonadales*	*Veillonellaceae*	*Dialister*	NS	↓	↓	↓	↓	NS
Firmicutes	*Clostridia*	*Clostridiales*	*Lachnospiraceae*	*Clostridium*	NS	↓	↓	NS	↓	↓
Bacteroidetes	*Bacteroidia*	*Bacteroidales*	*Paraprevotellaceae*	*CF231*	NS	↓	↓	NS	NS	NS
Firmicutes	*Clostridia*	*Clostridiales*	*Lachnospiraceae*	*Butyrivibrio*	NS	↓	↓	NS	NS	NS
Bacteroidetes	*Bacteroidia*	*Bacteroidales*	*Prevotellaceae*	*Prevotella*	NS	NS	NS	↓	↓	NS
Firmicutes	*Clostridia*	*Clostridiales*	*Lachnospiraceae*	*Blautia*	NS	NS	NS	↓	↓	NS
Proteobacteria	*Betaproteobacteria*	*Burkholderiales*	*Alcaligenaceae*	*Sutterella*	NS	NS	NS	↓	↓	NS
Firmicutes	*Clostridia*	*Clostridiales*	*Erysipelotrichaceae*	*Eubacterium*	NS	NS	NS	↓	NS	↑
Firmicutes	*Clostridia*	*Clostridiales*	*Lachnospiraceae*	*Dorea*	NS	NS	NS	NS	↓	↓
Firmicutes	*Clostridia*	*Clostridiales*	*Lachnospiraceae*	*Ruminococcus*	NS	NS	NS	NS	↓	↓

The table compares the change (increase or decrease) of fecal microbial taxa among the long-term alcohol drinking group (L), the short-term alcohol drinking group (S), and the control group (C), as calculated with LEfSe and MaAsLin2. The drinking (D) and abstinence (A) conditions are combined. A green arrow (**↑**) indicates a significantly higher relative abundance of a microbial taxon in a group of baboons compared to another group. A red arrow (**↓**) indicates a significantly lower relative abundance of a microbial taxon in a group of baboons compared to another group. Non-significant (NS) differences are also shown.

### Effect of chronic excessive alcohol drinking on fecal metabolome and on metabolic potential of the gut microbiome

**Fig. 5:**
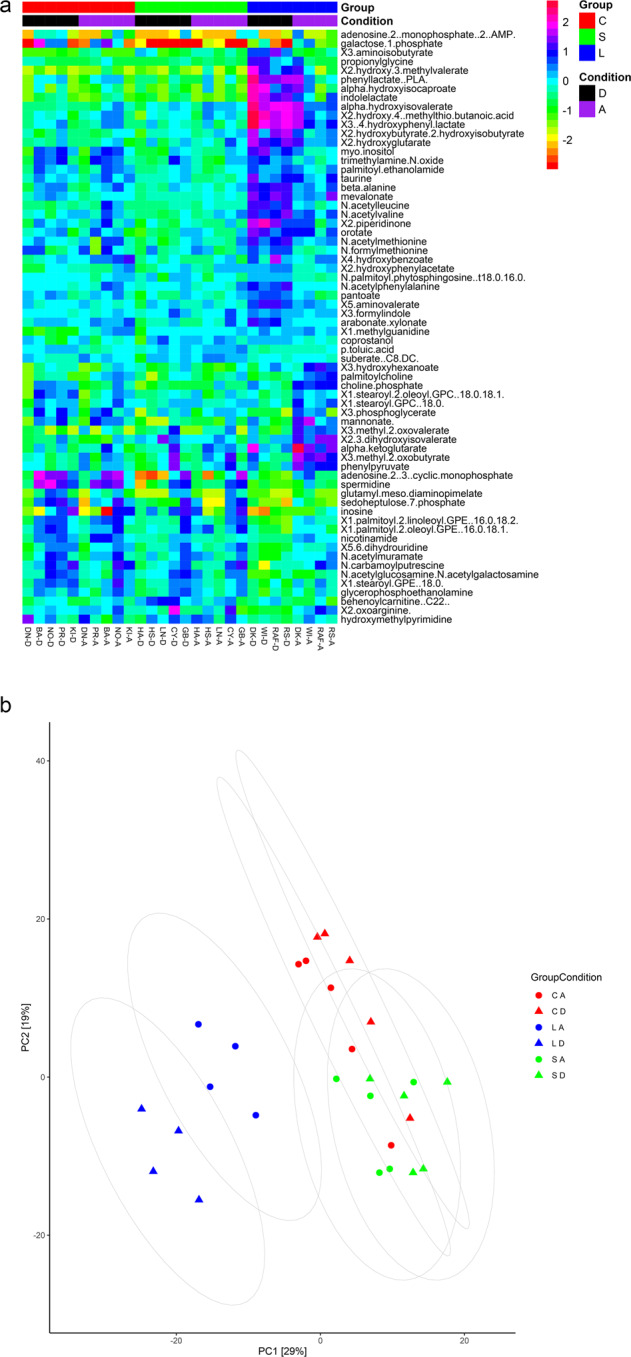
Effects of chronic excessive alcohol drinking on fecal metabolome. **a** Heatmap showing those metabolites (*N* = 63), out of the 534 identified, which achieved statistical significance (FDR-adjusted *p* < 0.01) in the group x condition interaction in the linear mixed models (LMMs). The long-term alcohol drinking group (L) is depicted in blue, the short-term alcohol drinking group (S) in green, and the control group (C) in red. The drinking condition (D) is depicted in black and the abstinence condition (A) in purple. **b** Score plot between the selected first two principal components (PCs) based on the output from the linear mixed models (LMMs). Group L is depicted in blue, group S in green, and group C in red. Triangles represent the drinking condition for each group (LD, SD, CD), circles represent the abstinence condition for each group (LA, SA, CA). Percent explained variance is reported in brackets on first (*x* axis) and second (*y* axis) principal component axis.

After fitting the linear mixed models (LMMs), 63/534 metabolites (12.2% of the total metabolites identified and analyzed) had a significant group x condition (GxC) interaction (FDR-adjusted *p* < 0.01) (Supplementary Table 5); they are visualized using a heatmap (Fig. 5a). Approximately two-thirds of these metabolites belong to either the amino acid (*N* = 23/63, 36.5%) or lipid (*N* = 19/63, 30.1%) super pathway. Another 16% belong to either the nucleotide (*N* = 7/63, 6.4%) or carbohydrate (*N* = 6/63, 9.5%) super pathway. The remainder are food components (*N* = 4/63, 11.1%), cofactors/vitamins (*N* = 3/63, 4.8%), or metabolites of the tricarboxylic acid cycle (TCA) (*N* = 1/63, 1.6%). The amino acid super pathway included acetylated adducts of the essential amino acids leucine and valine (N-acetylleucine, N-acetylvaline), as well as products of amino acid synthesis and degradation. The latter products were derived mainly from the aromatic amino acids tyrosine, phenylalanine, and tryptophan (*N* = 6/23), the branched-chain amino acids leucine, isoleucine, and valine (*N* = 5/23), the sulfur-containing amino acids methionine, cysteine, homocysteine, and taurine (*N* = 5/23), and tyrosine (3-(4-hydroxyphenyl)lactate). The lipid super pathway was comprised primarily of fatty acids and their derived metabolites (*N* = 6/19), together with phospholipid metabolites (*N* = 3/19), lysophospholipids (*N* = 2/19), phosphatidylethanolamines (*N* = 2/19), cholesterol derivatives (coprostanol), and endocannabinoids (palmitoyl ethanolamide). In the carbohydrate super pathway, most metabolites with a significant GxC interaction belonged to amino sugar (N-acetylglucosamine/N-acetylgalactosamine, N-acetylmuramate) and pentose metabolism (arabonate/xylonate, sedoheptulose-7-phosphate); one metabolite belonged to hexose metabolism (galactose 1-phosphate) and another one was an intermediate of metabolic pathways such as the Calvin cycle and glycolysis (3-phosphoglycerate). The seven metabolites in the nucleotide super pathway belonged to pyrimidine (*N* = 4/7) and purine metabolism (*N* = 3/7). A PCA based on the LMMs, with the GxC interaction term in it, revealed a clear separation between the L group and the other two groups, S and C; it also revealed a separation between conditions D and A within the L group (i.e., LD and LA). Differently, in the S and C groups, which displayed a closer metabolic profile, conditions D and A (i.e., SD, SA, CD, and CA) clustered together (Fig. 5b). The first principal component accounted for 29% of the variance, and the second principal component for 19%. It should be underlined that LMM fitting substantially reduced the variation of each metabolite signal, as shown by the comparison with a PCA performed before LMM fitting, where the first principal component accounted only for 21% of the variance, and the second principal component only for 14% (Supplementary Fig. 2).

**Fig. 6:**
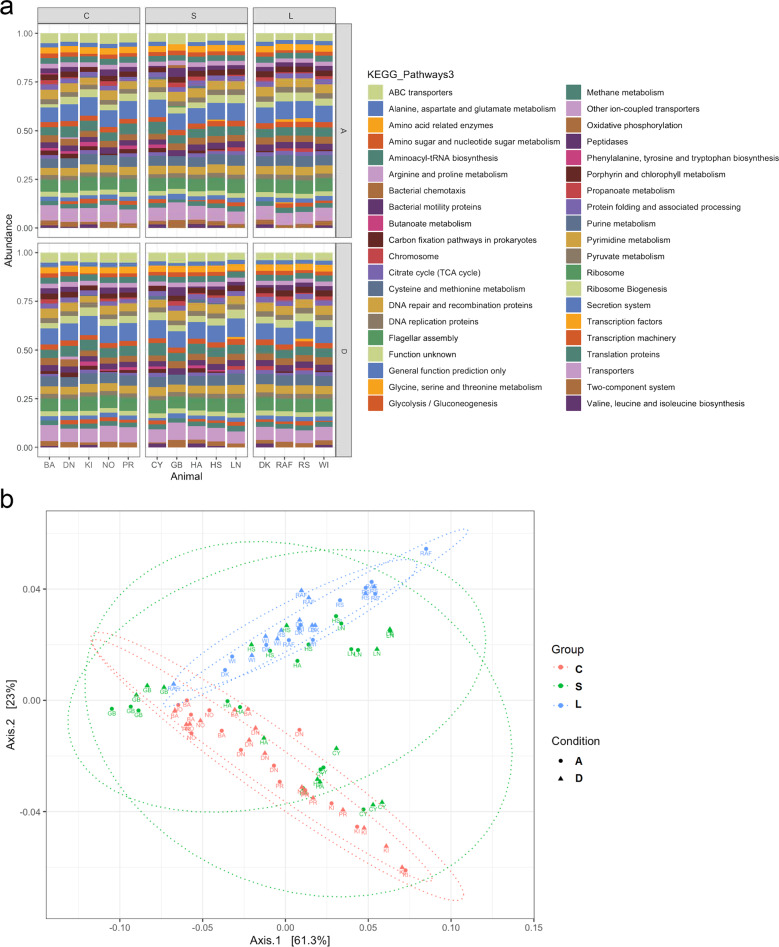
Effects of chronic excessive alcohol drinking on the functional role of the gut microbiome. **a** Phylogenetic investigation of communities by reconstruction of unobserved states (PICRUSt) comparing functional pathways among the long-term alcohol drinking group (L), the short-term alcohol drinking group (S), and the control group (C), as well as between the drinking (D) and abstinence (A) conditions. The baboons’ names are reported as acronyms. **b** Principal coordinates analysis (PCoA) plot comparing metagenome functional content of microbial taxa among group L (in blue), group S (in green), and group C (in red). Triangles represent condition D, circles represent condition A. The percent explained dissimilarity is reported in brackets on first (*x* axis) and second (*y* axis) principal coordinate axis. The distance between the triangles and circles, respectively, represents the difference in metabolic potential of the groups of baboons.

The results of the fecal metabolome analysis were backed up by the analysis of the metabolic potential of the gut microbiome using PICRUSt (Fig. 6a). We inferred the relative abundances of genes based on the abundance of bacterial OTUs in fecal samples, and compared them among the three groups L, S, and C and between the two conditions D and A. In condition D, the functional pathways related to aromatic amino acid metabolism, as well as those related to glycine, serine, and threonine metabolism, were more represented in the L group compared with the S and C groups. Pathways involved in purine biosynthesis were also enriched in the L group compared with the other two groups. Markers related to branched-chain amino acid metabolism showed an increase in the L and S groups in comparison with the C group. An upregulation of pathways involved in the TCA and amino sugar metabolism, as well as microbial carbon fixation pathways, such as the Calvin cycle, was observed in the L and S groups in comparison with the C group. An increase in KEGG orthologues associated with microbial chemotactic factors and motility proteins was observed in the L and S groups in comparison with the C group. In condition A, the pathways related to the TCA, as well as KEGG orthologues related to microbial chemotactic factors and motility proteins, showed a decrease in group L compared to the same group in condition D. The functional pathways related to branched-chain amino acid metabolism were suppressed in the L and S groups in condition A compared to the same groups in condition D. A PCoA (Bray Curtis Dissimilarity) based on PICRUSt data showed a clear separation between the L group and the other two groups, S and C, with the former group clustering in a different region of the plot (Fig. 6b). In the PCoA, conditions D and A overlapped within each group.

## Discussion

Changes in the fecal microbiome have been reported in rodents chronically exposed to alcohol [10–21], as well as in alcohol-dependent individuals [4–7, 28–36]. Two studies have investigated the effects of chronic alcohol self-administration on the gut bacteria in NHPs (male macaques) [44, 45]. Based on these studies, excessive alcohol consumption has been associated with quantitative and qualitative alterations of the gut microbiota (dysbiosis) [65]. A few animal [15, 20, 23, 24] and human studies [32, 38, 39] have performed fecal metabolomics to measure metabolites that reflect functional changes of the gut microbiota.

However, no studies of excessive alcohol drinking have examined the gut microbiome and metabolome in baboons, a species highly relevant from a translational standpoint. Here we analyze, for the first time, fecal microbial composition and function in an established baboon model of chronic excessive alcohol drinking in a controlled experimental setting, where animals self-administered alcohol ~1 g/kg and achieved BALs ≥ 0.08 g/dL within a 2-hour period (i.e., binge levels), daily, for years.

We found that fecal microbial alpha- (Shannon Diversity Index) and beta-diversity (Bray-Curtis Dissimilarity Matrix) were significantly lower in the L group compared with the S and C groups. The fecal microbial communities of the baboons did not differ significantly between conditions D and A, nor was there a significant interaction effect between the three groups and the two conditions.

The analysis of the fecal microbiota showed that the dysbiotic subpopulation of alcohol binge drinking baboons, i.e., group L, was characterized by a reduction of beneficial bacteria and an increase of potentially detrimental bacteria. This dysbiosis was characterized mainly by the lower relative abundance of the normal commensal Bacteroidetes phylum (*Prevotella* and *Parabacteroides* genera), the Lachnospiraceae family (*Blautia* genus), and genera such as *Faecalibacterium*, as well as the relative higher abundance of known opportunistic pathogens, such as members of the *Streptococcus* genus. Bacteroidetes phylum, which notably includes obligate anaerobes, could be decreased due to alcohol-induced oxidative stress [4]. Differently, *Streptococcus* is a facultative anaerobic genus and has a higher tolerance for oxidative stress [66], which could explain its higher abundance. Bacteria of the *Streptococcus* genus are generally considered harmful, as they are common pathogens responsible for bacterial infections [67]. Lachnospiraceae family has beneficial effects due to the ability to promote carbohydrate fermentation into short‐chain fatty acids (SCFAs) [68], which act as nutrients for the intestinal mucosa, modulators of gut pH, and suppressors of inflammation [69]. SCFAs are also produced by *Faecalibacterium* genus, which is typically found in a “healthy” gene-rich microbiome [70]. Our findings are consistent with some, but not all, of the previous rat [21], mouse [12, 13, 20], NHP [48], and human studies [4–7, 30, 34] on the effects of excessive alcohol use on fecal microbiota composition. It should be noted that some studies were carried out in rodents with experimental ALD [11, 15] or patients with AUD and ALD [29], which are known to have distinct gut microbiota profiles. The co-occurrence of ALD may act as a confounder and explain some of the observed discrepancies between findings. In addition, in our study, *Lactobacillus* genus increased from condition D to condition A in all three groups of baboons. A similar increase of this genus was observed in alcohol-dependent individuals after short-term abstinence [5]. The finding is noteworthy, given that this genus fulfills several beneficial health-promoting functions and is a commonly used probiotic [71].

Detailed compositional changes in the fecal microbiota of the baboons were investigated at different bacterial taxonomic levels. At the family level, LEfSe and MaAsLin2 algorithms showed significant differences among the three groups of baboons. Veillonellaceae, Prevotellaceae, and Lachnospiraceae decreased in the L and S groups compared with the C group, similarly to a study in alcohol-dependent individuals with high intestinal permeability [5]. Conversely, a study in male macaques reported an increase of Prevotellaceae following chronic alcohol self-administration [44]. Ruminococcaceae increased in group S, but not in group L. This microbial family, as well as *Parabacteroides* genus, increased in both rat and human alcohol drinkers—with rats exposed for 13 weeks to alcohol [14]. In our study, *Parabacteroides* increased in group S, but disappeared in group L.

At the genus level, LEfSe and MaAsLin2 results show that *Clostridium* gradually decreased from group C to group S to group L. A low abundance of this genus, which belongs to the Ruminococcaceae family, was observed in vaporized alcohol-exposed mice [13] and in alcohol-dependent individuals [4], especially in association with high intestinal permeability [5]. *Clostridium* is considered beneficial, as it’s populated by SCFA-producing species with anti-inflammatory properties [69]. *Lactobacillus* decreased in group S but increased in group L. An excess of *Lactobacillus* was observed in AUD patients with ALD [34], in whom alcohol-induced reductions in gut motility and pH may lead to fecal stasis and lumen bacterial proliferation [72]. This finding is in contrast with that of low *Lactobacillus* abundances in a number of rodent [11, 14, 15] and human studies [5, 29]. A decrease in *Faecalibacterium* was observed only in group L, possibly due to the long-term, chronic alcohol exposure needed to see this change. Indeed, a recent study revealed decreased abundances of *Faecalibacterium* in alcohol-dependent individuals with high intestinal permeability [5]. *Blautia* and *Dorea*, which belong to the Lachnospiraceae family, were less represented in the L group than in the S and C groups. A similar reduction of *Dorea* was observed in vaporized alcohol-exposed mice [13], whereas this genus, together with *Blautia*, was enriched in alcohol-dependent individuals with high intestinal permeability [5]. Reductions in *Dorea* have been linked with increases in pro-inflammatory cytokines [73].

In LEfSe, no significant differences between conditions D and A were found in taxa that exhibited >1% relative abundance. In MaAsLin2, only a few taxa differed significantly between the two conditions. The observed lack of major fecal microbial changes after the short period (3 days) of abstinence, which also holds true for microbial alpha- and beta-diversity, as discussed earlier, aligns with, and expands, previous NHP and human research. These studies showed: (a) only partial recovery of the gut microbiota of male macaques with long-term exposure to alcohol after a short period (5 days) of abstinence [45]; (b) the presence of gut dysbiosis in a subset of actively drinking and abstinent alcohol-dependent individuals, with no correlation between dysbiosis and duration of abstinence (4 weeks minimum) [4]; (c) no significant changes in the altered fecal microbial composition in alcohol-dependent individuals after short-term (3-week) abstinence, except for an increase, in some individuals, in members of the Ruminococcaceae family and the *Lactobacillus* and *Bifidobacterium* genera [5]; and (d) limited impact of short-term (3-week) abstinence on decreased fecal microbial alpha- and beta-diversity in AUD patients in the early stages of ALD [7]. Overall, these findings suggest that the effects of chronic excessive alcohol consumption on the gut microbiota are rather long-lasting and persist despite abstinent periods, at least short-term ones.

Recent work involving rodents has explored the effects of chronic alcohol administration on the gut microbiota metabolism. Alcohol-fed rats showed a significant increase in secondary BAs, carnitines, and steroid metabolites, a significant decrease in SCFAs, amino acids, and branched chain amino acids, as well as changes in primary BAs, fatty acids, lipids, and phenols compared with controls [23, 24]. In alcohol-fed mice saturated long-chain fatty acids decreased [15], whereas primary and secondary BAs, SCFAs, taurine, and serotonin increased [20] compared with controls. A study in male macaques reported an alteration in glycolysis during alcohol use (decreased glucose-6-phosphate and fructose-6-phosphate), as well as shifts in fatty acids after 5 days of abstinence [45]. In humans, chronic alcohol use has been associated with varying degrees of changes in fecal metabolites. The first fecal metabolomics study explored the effects of current and prior alcohol use on fecal BA profiles in alcohol-dependent patients, with and without ALD, compared with controls [32]. Current drinkers presented the highest levels of total BAs, secondary BAs, and secondary-to-primary BA ratio compared with the other groups, including controls. Primary BAs were not significantly different among groups. A subsequent study demonstrated changes in volatile organic compounds in the feces of alcohol-dependent patients in comparison with healthy controls, including a decrease in fatty acids with anti-oxidant properties, an increase in the oxidative stress marker tetradecane, and different levels of microbiota-related metabolites such as SCFAs and sulfides [38]. In a translational study of mice transplanted with fecal microbiota from alcohol-dependent individuals, with or without alcohol-associated hepatitis, secondary BAs increased with the increase of hepatitis severity [39].

In our study, fecal metabolic profiling highlighted a significant increase in microbiota-related metabolites involved in the aromatic amino acid pathways (e.g., N-acetylphenylalanine, phenyllactate, phenylpyruvate, indolelactate, 3-(4-hydroxyphenyl)lactate) in the L group compared with the S and C groups. Most of these metabolites had a significant group effect in the linear mixed-effects models in both conditions D and A. These changes suggest differences in gut microbial composition or activity between the alcohol drinking animals and controls. Yet, group S showed minimal differences from group C, possibly due to the longer duration of alcohol exposure needed to alter the normal gut microbiota. Notably, aromatic amino acids are the precursors of several neurotransmitters, i.e., tryptophan yields serotonin and catecholamines utilize tyrosine and phenylalanine as substrates [74]. Serotonin, dopamine, and norepinephrine are involved in the development and maintenance of AUD [75]. Serotonin has also a key role in the microbiota-gut-brain axis, acting as a neurotransmitter in both the central and the enteric nervous systems [76]. Microbiota-related metabolites of the methionine cycle (e.g., 2-hydroxy-4-(methylthio)butanoic acid, N-acetylmethionine, and N-formylmethionine) were significantly enriched in the L group, compared with the S and C groups, in condition D, but did not show significant differences among the three groups in condition A. Activation of the methionine cycle in the long-term excessive drinking animals is potentially detrimental, since in alcohol-treated mice s-adenosyl methionine, in combination with taurine and/or betaine, has been shown to prevent hepatotoxicity by acting on glutathione, a powerful antioxidant in the liver [77]. An increase in 2-hydroxybutyrate, a byproduct of methionine catabolism or glutathione synthesis (cysteine formation), was also found in the L group compared with the S and C groups. This metabolite is involved in alcohol-related oxidation and inflammation [78]. Products of tryptophan metabolism, including kynurenine and kynurenate, which are markers of intestinal inflammation [79], increased, although not significantly, in group L, compared with the S and C groups, in condition D. Microbiota-facilitated metabolites of the branched chain amino acids (e.g., 3-methyl-2-oxobutyrate, 3-methyl-2-oxovalerate, alpha-hydroxyisocaproate, and alpha-hydroxyisovalerate) increased significantly in the L group, compared with the S and C groups, mostly in condition A. Branched chain amino acids are essential amino acids that can be produced by the gut microbiota [80] and excessive alcohol use is associated with their decrease in the gut lumen [23]. The three days of abstinence (condition A) may be sufficient to reinstate their production by the gut microbiota of the long-term excessive drinking animals.

Varying degrees of changes in lipids were observed in the three groups of baboons. In the L group, compared with the S and C groups, long chain fatty acids and steroid metabolites increased, whereas acyl-carnitines and phosphatidylethanolamines decreased. These changes mainly occurred in condition D. The endocannabinoid palmitoyl ethanolamide showed a significant group effect in condition D, being significantly higher in the L group than in the other two groups. This finding is relevant, given the interactions between alcohol and the endocannabinoid system [81], and considering that the gut microbiota and the endocannabinoid system have an impact on gut barrier function and metabolic inflammation [82].

Additional fecal metabolome changes involved carbohydrate and central energy metabolism. In both conditions, the L group, compared to the other two groups, showed an increase in pentose (arabonate/xylonate) and TCA metabolites (alpha-ketoglutarate), suggesting elevated glycolysis and energy metabolism in the gut lumen in response to alcohol exposure [83].

An increase in N-acetylated amino acids (N-acetylleucine, N-acetylvaline) was observed exclusively in the L group in condition D, compared with the other two groups in the same condition. When acetylated proteins are broken down, the acetylated amino acids can be released into the circulation in their free form. Thus, the observed increase in fecal N-acetylated amino acids may be linked to gut mucosal damage mediated by long-term, chronic excessive alcohol drinking [84].

Further supporting the metabolomic data showing no significant difference in mucosal inflammation among the three groups of baboons, is also the apparent lack of meaningful differences in systemic inflammatory markers, although the small sample limits these conclusions and replication in larger samples is warranted.

Although only 10–20% of AUD patients develop ALD [85], their treatment is particularly challenging [86, 87]. Our work may be relevant for ongoing attempts to identify new treatments for these patients. A deeper investigation into gut dysbiosis and changes in microbiota-related metabolites could gain validity as an effective translational tool, to be used to restore the gut microbiota homeostasis to treat AUD and prevent ALD. Notably, in the present study, we did not observe any additional shifts in fecal microbial taxa that occur in association with ALD, beyond those occurring from excessive alcohol use per se. This is in line with the finding that our baboons presented with normal liver function.

A strength of our study is the use, for the first time, of a baboon model of long-term, chronic excessive alcohol drinking (up to 15 years) to investigate changes in the fecal microbiome and metabolome in controlled experimental conditions. The latter included consistent and reliable alcohol self-administration and BALs obtained via a CSR operant procedure, controlled food and caloric intake, regulated weight gain, identical housing and exposure to stimuli, and lack of confounders (e.g., geographic location, ethnicity, diet, cigarette smoking, illicit drugs, or medications). In contrast, these confounders are commonly and inevitably present in humans, resulting in high inter-individual variability, which can influence the shape of the gut microbiome [88]. Furthermore, baboons are phylogenetically close to humans and have comparable alcohol absorption and metabolism [42]. Importantly, the validity of this baboon CSR model has been extensively demonstrated in previous studies testing clinically-relevant alcohol behaviors and pharmacotherapies for AUD, including medications approved in clinical practice for AUD [46–48, 89–93]. Thus, this model bridges the gap between rodent and human data. Another strength of our study is the within-subject collection of fecal samples during both alcohol drinking and acute abstinence from alcohol, allowing us to test the effects of short-term abstinence on the fecal microbiome and metabolome after chronic alcohol exposure. Repeated measures also increase the validity and reliability of our findings. Most human studies take place at a single point in time, with participants varying in their last exposure to alcohol, although most of these studies are of chronic, heavy alcohol drinkers that often have developed ALD as a result.

A limitation of this study is the small sample, which limits statistical power, and may have led us to miss some alcohol-induced changes in the fecal microbiome or metabolome that have a small effect size. For example, a number of amino acids and fatty acids showed trends towards increased levels but did not reach statistical significance when correction for multiple testing was performed. Another limitation is that our sample included only males. Future efforts are needed to conduct research accounting for sex differences in the gut microbiota. Last, we did not analyze fecal microbial composition before administration of alcohol or Tang®, which could have provided us with insights into potential dysbiosis prior to exposure.

To conclude, in a baboon model of chronic excessive alcohol drinking, there were significant qualitative changes in the fecal microbiota of the long-term alcohol drinking group (up to 15 years), whereas relatively short-term alcohol drinking (~3 years) did not significantly alter it. Compared to Tang®, long-term and, less frequently, short-term alcohol drinking led to significant alterations in microbiota-related biochemicals, including metabolites of aromatic and branched chained amino acids, fatty acids, and pentose, as well as central energy metabolites and markers associated with intestinal tissue damage and oxidative stress. Collectively, changes in these pathways illustrate the emerging link between long-term excessive alcohol drinking, gut microbial dysbiosis, and associated fecal metabolite alterations. Dysbiosis was barely affected by acute forced abstinence from alcohol, whereas shifts in the fecal metabolome occurred when transitioning from the drinking to the abstinence condition. This observation suggests that changes in fecal microbiota function are more easily reversible than those of microbiota composition in response to acute abstinence from alcohol. We used a NHP model and a paradigm of excessive alcohol drinking that have never been used before to study the fecal microbiome and metabolome, and they are translationally relevant from a human perspective. While several confounders may be present in humans due to variability in lifestyle and exposure to environmental factors, our study was conducted in a controlled experimental setting, which allows us to have confidence that the observed changes in the fecal microbiome and metabolome can be attributed directly to the chronic excessive alcohol exposure. Future research will need to expand to other sections of the gastrointestinal tract [94], examine if more prolonged abstinence restores the fecal microbiome and metabolome, and determine if this line of research can contribute to the understanding and treatment of AUD.

## Supplementary information

Supplementary Material

Supplementary Fig. 1

Supplementary Fig. 2
