# Supplementary material for: Gut microbiome and metabolome in a non-human primate model of chronic excessive alcohol drinking
Source: Transl Psychiatry. 2021 Dec 1;11:609. doi: 10.1038/s41398-021-01728-6 (PMC8636625; doi:10.1038/s41398-021-01728-6)
Supplement: Supplementary file 1 — Supplementary Material [file 41398_2021_1728_MOESM1_ESM.docx]

**Supplementary Methods**

**Chained Schedule of Reinforcement (CSR) Operant Procedure**

Each baboon self-administered alcohol under a three-component chained schedule of reinforcement (CSR), as described in detail previously^1,2^. This procedure allows evaluation of responding in the presence of alcohol-related cues that is maintained by conditioned reinforcement (i.e., responding that produces access to alcohol or “seeking”), as well as alcohol self-administration and consumption within the same session. The start of a session was signaled by a 3-sec tone and onset of Component 1. During Component 1, a red cue light was illuminated for 20 min and all responses (i.e., lever presses; drinkometer contacts) were recorded but had no programmed consequence. Component 2 was signaled by the illumination of a yellow cue light and consisted of two links. During the first link, the jewel light over the left lever was turned on, and an alternate fixed-interval (FI) 10-min, fixed-time (FT) 20-min schedule was in effect on the left lever. The first link ended either a) with the first response on the left lever after 10 min elapsed or b) automatically after 20 min, whichever occurred first. During the second link, the jewel light over the left lever flashed and a fixed-ratio (FR) schedule was in effect on the left lever. Completion of the FR response requirement (FR10) ended Component 2; the yellow cue light and the jewel light were turned off, and Component 3 was initiated. Failure to complete the FR response requirement in Component 2 within 90 min resulted in the termination of the session. During Component 3, the blue cue light was illuminated, the jewel light over the right lever was illuminated, and the opportunity to orally self-administer alcohol was available according to a FR 10 schedule on the right lever. Completion of each FR turned the jewel light off and turned on the white lights on the drinkometer faceplate, indicating drink availability. Contact with the drinkometer spout turned off the white lights, turned on the green lights on the drinkometer faceplate, and operated a solenoid valve that delivered fluid for five seconds (~30 ml) or the duration of spout contact, whichever came first. This was defined as a single drink. Following each drink, all drinkometer lights were turned off and the jewel light over the right lever was again illuminated. Component 3 ended after 120 min and all programmed stimuli were turned off, with alcohol available only in the final component. This procedure allows for examination of responding in the presence of alcohol-related cues that is maintained by conditioned reinforcement (i.e., responding that produces access to the day's supply of alcohol or “seeking”), as well as alcohol self-administration and consumption within the same session.

**Metabolomics Sample Accessioning**

Following receipt, samples were inventoried and immediately stored at -80^o^C. Each sample received was accessioned into the Metabolon Laboratory Information Management System (LIMS) and was assigned by the LIMS a unique identifier that was associated with the original source identifier only. This identifier was used to track the handling, tasks, and results of the samples (and all derived aliquots). All portions of any sample were automatically assigned their own unique identifiers by the LIMS when a new task was created; the relationship of these samples was also tracked. All samples were maintained at -80^o^C until processed.

**Metabolomics Sample Preparation**

Samples were prepared using the automated MicroLab STAR® system (Hamilton Company, Franklin, MA, USA) and extracted using Metabolon’s standard solvent extraction method, as described in detail previously^3^. Several recovery standards were added prior to the extraction process for quality control [QC] purposes. To remove proteins, dissociate small molecules bound to proteins or trapped in the precipitated protein matrix, and to recover chemically diverse metabolites, proteins were precipitated with methanol under vigorous shaking for 2 minutes (Glen Mills GenoGrinder 2000) followed by centrifugation. The resulting extract was divided into five fractions: three for analysis by untargeted reverse phase ultra-performance liquid chromatography-tandem mass spectrometry (RP-UPLC-MS/MS, Waters ACQUITY, Milford, MA, USA) with electrospray ionization (ESI, Thermo Scientific, Waltham, MA, USA) in both positive (two fractions) and negative (one fraction) modes, one for analysis by hydrophilic interaction chromatography (HILIC-UPLC-MS/MS-ESI) with negative ion mode, and a last one was reserved for backup. Samples were placed briefly on a Zymark TurboVap® (McKinley Scientific, Sparta, NJ, USA) to remove the organic solvent. The sample extracts were stored overnight under nitrogen before preparation for analysis.

**Metabolomics Quality Control (QC)**

Several types of QC samples were analyzed in concert with the experimental samples, as described in detail previously^4,5^: a pooled matrix sample generated by taking a small volume of each experimental sample (i.e., client matrix or CMTRX) and a pool of well-characterized human plasma maintained by Metabolon (i.e., matrix or MTRX) served as technical replicates throughout the data set (to ensure that all aspects of the Metabolon process were operating within specifications and to assess the effect of a non-plasma matrix on the Metabolon process and distinguish biological variability from process variability, respectively); ultra-pure water samples served as process blanks (to assess the contribution to compound signals from the process); and solvents used during the extraction process served as solvent blanks (to segregate contamination sources in the extraction). A cocktail of QC standards that were carefully chosen so as not to interfere with the measurement of endogenous compounds were spiked into every analyzed sample, allowing instrument performance monitoring and aiding chromatographic alignment. QC standards included a recovery standard (to assess variability and verify performance of extraction and instrumentation) and an internal standard (to assess variability and performance of instrumentation). Instrument variability was determined by calculating the median relative standard deviation (RSD) for the standards that were added to each sample prior to injection into the mass spectrometers. Overall process variability was determined by calculating the median RSD for all endogenous metabolites (i.e., non-instrument standards) present in 100% of the pooled matrix samples. Experimental samples were randomized across the platform run with QC samples spaced evenly among the injections.

**Metabolomics Ultra Performance Liquid Chromatography-Tandem Mass Spectrometry (UPLC/MS/MS)**

All methods used a Waters ACQUITY UPLC and a Thermo Scientific Q-Exactive high resolution/accurate mass spectrometer interfaced with a heated electrospray ionization (HESI-II) source and an Orbitrap mass analyzer operated at 35,000 mass resolution. The sample extract was dried, then reconstituted in solvents compatible to each of the four aforementioned methods of analysis. Each reconstitution solvent contained a series of standards at fixed concentrations to ensure injection and chromatographic consistency. At the UPLC/MS/MS, one aliquot was analyzed using acidic positive ion conditions, chromatographically optimized for more hydrophilic compounds. In this method, the extract was gradient eluted from a C18 column (Waters UPLC BEH C18-2.1x100 mm, 1.7 µm) using water and alcohol, containing 0.05% perfluoropentanoic acid (PFPA) and 0.1% formic acid (FA). Another aliquot was also analyzed using acidic positive ion conditions; however, it was chromatographically optimized for more hydrophobic compounds. In this method, the extract was gradient eluted from the same C18 column using methanol, acetonitrile, water, 0.05% PFPA and 0.01% FA, and was operated at an overall higher organic content. Another aliquot was analyzed using basic negative ion optimized conditions using a separate dedicated C18 column. The basic extracts were gradient eluted from the column using methanol and water with 6.5 mM ammonium bicarbonate at pH 8. The fourth aliquot was analyzed via negative ionization following elution from a HILIC column (Waters UPLC BEH Amide 2.1x150 mm, 1.7 µm) using a gradient consisting of water and acetonitrile with 10mM ammonium formate, pH 10.8. The mass spectra (MS) analysis alternated between MS and data-dependent MS^n^ scans using dynamic exclusion. The scan range varied slightly between methods but covered 70-1000 m/z. Raw data files were archived and extracted as described below.

**Metabolomics Data Extraction and Compound Identification**

Raw data was extracted, peak-identified, and QC processed using Metabolon’s hardware and software^4,5^. These systems are built on a web-service platform utilizing Microsoft’s .NET technologies, which run on high-performance application servers and fiber-channel storage arrays in clusters to provide active failover and load-balancing. Compounds were identified by comparison to library entries of purified standards or recurrent unknown entities. Metabolon maintains a library based on authenticated standards that contains the retention time/index (RI), mass to charge ratio (*m/z)*, and chromatographic data (including MS/MS spectral data) of all molecules present in the library. Biochemical identifications are based on three criteria: retention index within a narrow window of the proposed identification, accurate mass match to the library +/- 10 ppm, and the MS/MS forward and reverse scores between the experimental data and authentic standards. The MS/MS scores are based on a comparison of the ions present in the experimental spectrum to the ions present in the library spectrum. While there may be similarities between these molecules based on one of these factors, the use of all three of them allows to distinguish and differentiate biochemicals. More than 3,300 commercially available purified standard compounds have been acquired and registered into LIMS for analysis on all platforms for determination of their analytical characteristics. Additional mass spectral entries have been created for structurally unnamed biochemicals, which have been identified by virtue of their recurrent nature (both chromatographic and mass spectral). These compounds have the potential to be identified by future acquisition of a matching purified standard or by classical structural analysis.

**Metabolomics Data Curation**

A variety of curation procedures were carried out to ensure that a high-quality dataset was made available for statistical analysis and data interpretation. The QC and curation processes were designed to ensure accurate and consistent identification of true chemical entities, and to remove those representing process artifacts, mis-assignments, and background noise. Process artifacts were defined as biochemicals present in the biological samples at ≤3 times the level in the water process blanks. Metabolon data analysts use proprietary visualization and interpretation software to confirm the consistency of peak identification among the various samples. Library matches for each compound were checked for each sample and corrected if necessary.

**Cortisol and Cytokine Measurement**

Blood for plasma cortisol and serum cytokines was collected between 10:00-11:00 AM directly from the saphenous vein. Blood samples to be used for plasma cortisol analysis were collected into K2EDTA BD Vacutainer tubes treated with an enzymatic inhibitor (Cat # DPP40, Millipore Sigma, Billerica, MA, USA) and placed immediately on wet ice. Tubes were centrifuged at 1700 x g for 15 minutes at 4°C within 15-30 minutes of sample collection. Blood samples used for serum cytokine analysis were collected into BD Vacutainer tubes with a gel separator and allowed to clot at room temperature for 30 minutes before centrifuging at 1000 x g for 10 minutes at 25°C. Plasma and serum samples were stored at -80°C until analysis. Plasma cortisol was assayed using a commercially available competitive enzyme-linked immunoassay (ELISA) (Item no. 500360, Caymen Chemical, Ann Arbor, MI, USA) according to manufacturer instructions. Samples were diluted 1:2000 prior to assay and absorbance was read at 405 nm on a CLARIOstar Plus (BMG Labtech, Cary, NC, USA) microplate reader. The standard curve was fit with a 4-parameter logistic equation using GraphPad Prism version 9 (GraphPad Software, Inc. San Diego, CA, USA). Assay range and sensitivity as reported by the manufacturer was 6.6-4000 pg/mL and 35 pg/mL, respectively. Serum cytokines were analyzed using a commercially available non-human primate cytokine array (Cat# QAN-CYT-1, RayBiotech, Peachtree Corners, GA, USA) according to manufacturer instructions. Briefly, slides were warmed to room temperature and then allowed to air dry for 2 hours. 100 μL of sample diluent was then added to each well and allowed to incubate for 30 min at 25°C with gentle rocking to block slides. 100 μL of standard or sample was added to wells and slides were allowed to incubate overnight at 4°C. Slides were then washed prior to adding 80 μL of detection antibody to each well and incubating again overnight at 4°C. Lastly, slides were washed and 80 μL of Cy3 equivalent dye-conjugated streptavidin was added to each well and allowed to incubate with gentle rocking for 1 hour at 25°C. Slides were then washed and completely dried according to manufacturer instructions. Fluorescence intensity of each well array was extracted using a microarray laser scanner at RayBiotech Service Department (Peachtree Corners, GA) and concentration was determined by fitting a log-log or linear regression curve for each analyte. Inter-assay % coefficient of variation (CV) is <20%, as reported by the manufacturer.

**References**

1. Weerts, E.M., Goodwin, A.K., Kaminski, B.J. & Hienz, R.D. Environmental cues, alcohol seeking, and consumption in baboons: Effects of response requirement and duration of alcohol abstinence. *Alcohol Clin. Exp. Res.* **30**, 2026–2036 (2006).
2. Kaminski, B.J., Goodwin, A.K., Wand, G. & Weerts, E.M. Dissociation of alcohol‐seeking and consumption under a chained schedule of oral alcohol reinforcement in baboons. *Alcohol Clin. Exp. Res.* **32**, 1014–1022 (2008).
3. Evans, A.M. Bridgewater, B., Liu, Q., Mitchell, M., Robinson, R., Dai, H. et al. High-resolution mass spectrometry improves data quantity and quality as compared to unit mass resolution mass spectrometry in high-throughput profiling metabolomics. *Metabolomics* **4**, 132 (2014).
4. Evans, A.M., DeHaven, C.D., Barrett, T., Mitchell, M. & Milgram, E. Integrated, nontargeted ultrahigh performance liquid chromatography/electrospray ionization tandem mass spectrometry platform for the identification and relative quantification of the small-molecule complement of biological systems. *Anal. Chem.* **81**, 6656–6667 (2009).
5. DeHaven, C.D., Evans, A., Dai, H. & Lawton, K. Organization of GC/MS and LC/MS metabolomics data into chemical libraries. *J. Cheminformatics* **2**, 9 (2010).

**Supplementary** **Fig. 1**. **Distribution of sample sequencing depth**. The total number of reads (read counts) obtained from a sequencing run is shown.

**Supplementary** **Fig. 2**. **Principal component analysis before linear mixed model (LMM) fitting**. Scores plot between the selected first two principal components (PCs) before fitting the linear mixed models (LMMs). The long-term alcohol drinking group (L) is depicted in blue, the short-term alcohol drinking group (S) in green, and the control group (C) in red. Triangles represent the drinking condition for each group (LD, SD, CD), circles represent the abstinence condition for each group (LA, SA, CA). Percent explained variance is reported in brackets on first (x-axis) and second (y-axis) principal component axis.

| **Variable** | **Long-term alcohol drinking (*N*=4)** | **Short-term alcohol drinking (*N*=5)** | **Control (*N*=5)** | ***p*-value** |
| --- | --- | --- | --- | --- |
| IL-1β in pg/mL, median (IQR) | 64.7 (67.0) | 27.9 (96.2) | 10.5 (76.9) | 0.495 |
| IL-4 in pg/mL, median (IQR) | 9.2 (35.4) | 45.4 (74.4) | 98.4 (98.1) | 0.049 |
| IL-5 in pg/mL, median (IQR) | 30.4 (29.1) | 18.6 (26.6) | 4.1 (18.1) | 0.141 |
| IL-6 in pg/mL, median (IQR) | 371.2 (423.8) | 181.8 (499.0) | 37.1 (223.8) | 0.112 |
| IL-12p70 in pg/mL, median (IQR) | 549.8 (1616.0) | 350.4 (1782.0) | 381.4 (1046.0) | 0.588 |
| IL-15 in pg/mL, median (IQR) | 754.4 (2215.0) | 388.1 (767.0) | 68.5 (768.0) | 0.300 |
| IL-16 in pg/mL, median (IQR) | 2606.8 (2124.0) | 2557.1 (2973.0) | 328.3 (3971.0) | 0.379 |
| IFN-γ in pg/mL, median (IQR) | 377.4 (520.0) | 352.20 (338.3) | 58.5 (193.9) | 0.115 |
| TNF-α in pg/mL, median (IQR) | 88.4 (104.7) | 63.6 (82.4) | 7.7 (57.6) | 0.161 |
| GM-CSF in pg/mL, median (IQR) | 77.0 (64.7) | 38.7 (49.8) | 9.7 (27.1) | 0.056 |
| Cortisol in µg/L, median (IQR) | 224.0 (109.5) | 160.3 (112.1) | 98.3 (50.7) | 0.061 |

**Supplementary Table 1. Baboons’ cytokine and cortisol levels**. Medians and interquartile ranges (IQR) are shown for each variable. Kruskal-Wallis test was used to compare the median values of each variable among the three groups of baboons, i.e., the long-term alcohol drinking group, the short-term alcohol drinking group, and the control group. The reported range of each cytokine was as follows: interleukin (IL)-1β: 1.7-370.4 pg/mL, IL-4: 0.9-370.4 pg/mL, IL-5: 0.4-185.2 pg/mL, IL-6: 4.1-3703.7 pg/mL, IL-12p70: 26.3-20,000 pg/mL, IL-15: 6.0-40,000 pg/mL, IL-16: 49.2-12,345.7 pg/mL, interferon-gamma (IFNγ): 4.0-1,851.9 pg/mL, tumor necrosis factor-alpha (TNFα): 1.1-246.9 pg/mL, granulocyte-macrophage colony-stimulating factor (GM-CSF): 1.3-370.4 pg/mL. The reported range of cortisol was 6.6-4000 pg/mL.

| **Source of variation** | **Degrees of freedom** | **Sum of squares** | **Mean sum of squares** | ***F*** | **R^2^** | ***P*^a^ (perm)** |
| --- | --- | --- | --- | --- | --- | --- |
| Groups: L, S, C | 2 | 3.462 | 1.731 | 10.760 | 0.220 | 0.001 |
| Condition: D, A | 1 | 0.098 | 0.098 | 0.612 | 0.006 | 0.850 |
| Group x Condition | 2 | 0.130 | 0.065 | 0.403 | 0.008 | 0.999 |
| Residuals | 75 | 12.754 | 0.161 |  | 0.766 |  |
| Total | 80 | 15.754 |  |  | 1.000 |  |

**Supplementary Table 2**. **Permutational ANOVA (PERMANOVA) testing whether the microbial communities had different centroids based on exposure or genotype**. PERMANOVA (Adonis) results for the long-term alcohol drinking group (L) *vs.* the short-term alcohol drinking group (S) *vs.* the control group (C) based on Bray-Curtis Dissimilarity Index, as well as for the drinking (D) condition *vs*. the abstinence (A) condition and for the group x condition interaction. **^a^***P*-values are based on 999 permutations.

| **Metadata** | **Feature** | **Value** | **Coefficient** | **Standard error** | ***p*-value** |
| --- | --- | --- | --- | --- | --- |
| Group | Streptococcaceae_Streptococcus | C | -0.08034 | 0.021463 | 0.003249 |
| Group | Streptococcaceae_Streptococcus | S | -0.06154 | 0.021463 | 0.015317 |
| Group | Lactobacillaceae_Lactobacillus | C | -0.13497 | 0.049887 | 0.020453 |
| Group | Lactobacillaceae_Lactobacillus | S | -0.18009 | 0.049887 | 0.004098 |
| Group | Veillonellaceae_Dialister | C | 0.016761 | 0.004359 | 0.002722 |
| Group | Veillonellaceae_Dialister | S | 0.012695 | 0.004359 | 0.014129 |
| Group | Prevotellaceae_Prevotella | C | 0.080001 | 0.034532 | 0.040813 |
| Group | Prevotellaceae_Prevotella | S | 0.002425 | 0.000936 | 0.025146 |
| Group | Alcaligenaceae_Sutterella | C | 0.002887 | 0.000978 | 0.013171 |
| Group | Alcaligenaceae_Sutterella | S | 0.002547 | 0.000978 | 0.024534 |
| Group | Lachnospiraceae_Blautia | C | 0.018409 | 0.008315 | 0.048882 |
| Group | Lachnospiraceae_Blautia | S | 0.020137 | 0.008315 | 0.033906 |
| Group | Lachnospiraceae_Oribacterium | S | 0.009901 | 0.003103 | 0.00859 |
| Group | Lachnospiraceae_Butyrivibrio | C | 0.004902 | 0.001576 | 0.00992 |
| Group | Lachnospiraceae_Dorea | C | 0.003706 | 0.001563 | 0.037122 |
| Group | Lachnospiraceae_Ruminococcus | C | 0.006192 | 0.002741 | 0.04519 |
| Group | Ruminococcaceae_Faecalibacterium | S | 0.025933 | 0.0071 | 0.003805 |
| Group | Porphyromonadaceae_Parabacteroides | S | 0.010264 | 0.004427 | 0.040693 |

**Supplementary Table 3**. **Effects of chronic excessive alcohol drinking on fecal microbial composition in the three groups of baboons based on the multivariate microbial association by linear models (MaAsLin2)**. The table shows the significant changes (increase or decrease) of fecal microbial taxa in the short-term alcohol drinking group (S) and in the control group (C), respectively, compared with the long-term alcohol drinking group (L), as measured by MaAsLin2. The drinking (D) and abstinence (A) conditions are combined.

| **Metadata** | **Feature** | **Value** | **Coefficient** | **Standard error** | ***p*-value** |
| --- | --- | --- | --- | --- | --- |
| Condition | Lachnospiraceae_Oribacterium | D | 0.001562 | 0.000515 | 0.009565 |
| Condition | Ruminococcaceae_Oscillospira | D | 0.004195 | 0.001485 | 0.014333 |
| Condition | Porphyromonadaceae_Parabacteroides | D | 0.002102 | 0.000774 | 0.017627 |
| Condition | Lachnospiraceae_Roseburia | D | 0.002995 | 0.001181 | 0.024887 |
| Condition | Lactobacillaceae_Lactobacillus | D | -0.03513 | 0.015027 | 0.036063 |
| Condition | Coriobacteriaceae_Collinsella | D | 0.002476 | 0.001107 | 0.043422 |

**Supplementary Table 4**. **Effects of chronic excessive alcohol drinking on fecal microbial composition in the drinking and abstinence condition** **based on the multivariate microbial association by linear models (MaAsLin2)**. The table shows the significant changes (increase or decrease) of fecal microbial taxa in the drinking (D) condition compared with the abstinence (A) condition, as measured by MaAsLin2. The groups of baboons, i.e., the long-term alcohol drinking group (L), the short-term alcohol drinking group (S), and the control group (C), are combined.

| **Super pathway** | **Pathway** | **Biochemical Name** | **Group x condition interaction effect (FDR *p*-value)** | **Group effect in D (*p*-value)** | **Group effect in A (*p*-value)** | **D** | | | **A** | | |
| --- | --- | --- | --- | --- | --- | --- | --- | --- | --- | --- | --- |
|  |  |  |  |  |  | **L *vs*. S** | **L *vs*. C** | **S *vs*. C** | **L vs. S** | **L *vs*. C** | **S *vs*. C** |
| Amino acid | Cystathionine metabolism | 2-hydroxybutyrate | 0.0007 | 0.0007 | 0.0501 | **↑** | **↑** | NS | **↑** | NS | NS |
|  | Guanidino and acetamido metabolism | 1-methylguanidine | 0.0001 | <0.0001 | 0.0871 | NS | **↑** | **↑** | NS | NS | NS |
|  | Leucine, isoleucine, and valine metabolism | 2-hydroxy-3-methylvalerate | 0.0017 | <0.0001 | 0.2844 | **↑** | **↑** | NS | NS | NS | NS |
|  |  | 3-methyl-2-oxobutyrate | 0.0033 | 0.5636 | 0.0005 | NS | NS | NS | **↑** | **↑** | NS |
|  |  | 3-methyl-2-oxovalerate | 0.0009 | 0.1451 | 0.0021 | NS | NS | NS | **↑** | **↑** | NS |
|  |  | N-acetylleucine | 0.0002 | <0.0001 | 0.3106 | **↑** | **↑** | NS | NS | NS | NS |
|  |  | N-acetylvaline | 0.0031 | <0.0001 | 0.2219 | **↑** | **↑** | NS | NS | NS | NS |
|  |  | alpha-hydroxyisocaproate | 0.0034 | 0.0003 | 0.2846 | **↑** | **↑** | NS | NS | NS | NS |
|  |  | alpha-hydroxyisovalerate | 0.0001 | <0.0001 | 0.015 | **↑** | **↑** | NS | **↑** | **↑** | NS |
|  | Lysine metabolism | 5-aminovalerate | 0.0002 | <0.0001 | 0.006 | **↑** | **↑** | NS | **↑** | **↑** | NS |
|  | Methionine, cysteine, S-adenosyl methionine (SAM), and taurine metabolism | 2-hydroxy-4-(methylthio)butanoic acid | 0.0000 | <0.0001 | 0.104 | **↑** | **↑** | NS | **↑** | NS | NS |
|  |  | N-acetylmethionine | 0.0007 | <0.0001 | 0.214 | **↑** | **↑** | **↑** | NS | NS | NS |
|  |  | N-formylmethionine | 0.0078 | 0.0078 | 0.1337 | **↑** | **↑** | NS | NS | NS | NS |
|  |  | taurine | 0.0066 | 0.0092 | 0.0016 | **↑** | **↑** | NS | **↑** | NS | NS |
|  | Phenylalanine metabolism | 2-hydroxyphenylacetate | 0.0034 | 0.0009 | 0.9004 | **↑** | **↑** | NS | NS | NS | NS |
|  |  | N-acetylphenylalanine | 0.0014 | 0.0002 | 0.4648 | **↑** | **↑** | NS | NS | NS | NS |
|  |  | phenyllactate | 0.0015 | <0.0001 | 0.122 | **↑** | **↑** | NS | **↑** | NS | NS |
|  |  | phenylpyruvate | 0.0050 | 0.881 | 0.001 | NS | NS | NS | **↑** | **↑** | NS |
|  | Polyamine metabolism | N-carbamoylputrescine | 0.0094 | 0.0337 | 0.92 | NS | NS | NS | NS | NS | NS |
|  |  | spermidine | 0.0061 | 0.0009 | 0.0377 | NS | **↓** | **↓** | NS | **↓** | NS |
|  | Tryptophan metabolism | indolelactate | 0.0079 | <0.0001 | 0.0313 | **↑** | **↑** | NS | **↑** | **↑** | NS |
|  | Tyrosine metabolism | 3-(4-hydroxyphenyl)lactate | 0.0002 | <0.0001 | 0.006 | **↑** | **↑** | NS | **↑** | **↑** | NS |
|  | Urea cycle; arginine and proline metabolism | 2-oxoarginine | 0.0023 | <0.0001 | 0.4539 | **↓** | **↓** | NS | NS | NS | NS |
| Carbohydrate | Amino sugar metabolism | N-acetylglucosamine/N-acetylgalactosamine | 0.0023 | 0.0008 | 0.0332 | **↓** | **↓** | NS | **↓** | NS | NS |
|  |  | N-acetylmuramate | 0.0037 | 0.0468 | 0.9867 | **↓** | **↓** | NS | NS | NS | NS |
|  | Fructose, mannose, and galactose metabolism | galactose 1-phosphate | 0.0066 | 0.0416 | 0.0037 | NS | NS | **↓** | NS | NS | **↓** |
|  | Glycolysis, gluconeogenesis, and pyruvate metabolism | 3-phosphoglycerate | 0.0044 | 0.0062 | 0.7349 | NS | **↓** | NS | NS | NS | NS |
|  | Pentose metabolism | arabonate/xylonate | 0.0050 | 0.0031 | 0.0952 | **↑** | **↑** | **↑** | NS | **↑** | NS |
|  | Pentose phosphate pathway | sedoheptulose-7-phosphate | 0.0023 | 0.0041 | 0.4825 | **↓** | **↓** | NS | NS | NS | NS |
| Cofactors and vitamins | Nicotinate and nicotinamide metabolism | nicotinamide | 0.0029 | 0.0251 | 0.0138 | NS | NS | NS | **↑** | NS | NS |
|  | Pantothenate and CoA metabolism | pantoate | 0.0034 | 0.0003 | 0.1986 | **↑** | **↑** | NS | NS | NS | NS |
|  | Thiamine metabolism | hydroxymethylpyrimidine | 0.0094 | 0.024 | 0.3548 | **↓** | NS | NS | NS | NS | NS |
| Energy | Tricarboxylic acid (TCA) cycle | alpha-ketoglutarate | 0.0037 | 0.1698 | 0.0091 | NS | NS | NS | **↑** | **↑** | NS |
| Lipid | Dihydroceramides | N-palmitoyl-phytosphingosine (t18:0/16:0) | 0.0033 | 0.0178 | 0.1337 | NS | **↑** | NS | NS | **↑** | NS |
|  | Endocannabinoid | palmitoyl ethanolamide | 0.0094 | 0.0125 | 0.1122 | **↑** | **↑** | NS | NS | NS | NS |
|  | Fatty acid metabolism (acyl carnitine, long chain saturated) | behenoylcarnitine (C22) | 0.0070 | 0.0038 | 0.1097 | **↓** | NS | **↓** | **↓** | NS | NS |
|  | Fatty acid metabolism (acyl choline) | palmitoylcholine | 0.0033 | 0.9185 | 0.013 | NS | NS | NS | **↑** | NS | NS |
|  | Fatty acid metabolism (also branched-chain amino acids metabolism) | propionylglycine | 0.0098 | 0.0054 | 0.655 | **↑** | **↑** | NS | NS | NS | NS |
|  | Fatty acid, monohydroxy | 3-hydroxyhexanoate | 0.0005 | 0.4412 | 0.007 | NS | NS | NS | **↑** | **↑** | NS |
|  | Fatty acid, dicarboxylate | 2-hydroxyglutarate | 0.0042 | <0.0001 | 0.0158 | **↑** | **↑** | NS | **↑** | **↑** | NS |
|  |  | suberate (C8-DC) | 0.0065 | 0.9481 | 0.0227 | NS | NS | NS | **↓** | NS | NS |
|  | Inositol metabolism | myo-inositol | 0.0014 | 0.0006 | 0.2829 | **↑** | NS | NS | NS | NS | NS |
|  | Lysophospholipid | 1-stearoyl-GPC (18:0) | 0.0099 | 0.7287 | 0.0701 | NS | NS | NS | **↑** | NS | NS |
|  |  | 1-stearoyl-GPE (18:0) | 0.0061 | 0.0131 | 0.7476 | NS | **↑** | NS | NS | NS | NS |
|  | Mevalonate metabolism | mevalonate | 0.0033 | <0.0001 | 0.0204 | **↑** | **↑** | NS | **↑** | **↑** | NS |
|  | Phosphatidylcholine (PC) | 1-stearoyl-2-oleoyl-GPC (18:0/18:1) | 0.0031 | 0.4578 | 0.1233 | NS | NS | NS | NS | NS | NS |
|  | Phosphatidylethanolamine (PE) | 1-palmitoyl-2-linoleoyl-GPE (16:0/18:2) | 0.0021 | 0.001 | 0.215 | **↓** | **↓** | NS | NS | NS | NS |
|  |  | 1-palmitoyl-2-oleoyl-GPE (16:0/18:1) | 0.0022 | 0.012 | 0.2179 | **↓** | **↓** | NS | NS | NS | NS |
|  | Phospholipid metabolism | choline phosphate | 0.0079 | 0.0131 | <0.0001 | **↑** | NS | **↑** | **↑** | **↑** | NS |
|  |  | glycerophosphoethanolamine | 0.0080 | 0.0047 | 0.5863 | **↓** | **↓** | NS | NS | NS | NS |
|  |  | trimethylamine N-oxide | 0.0061 | 0.157 | 0.6507 | NS | NS | NS | NS | NS | NS |
|  | Sterol | coprostanol | 0.0049 | 0.0358 | 0.0765 | **↑** | **↑** | NS | NS | NS | NS |
| Nucleotide | Purine metabolism, adenine containing | adenosine 2'-monophosphate (2'-AMP) | 0.0012 | 0.3625 | 0.5452 | NS | NS | NS | NS | NS | NS |
|  |  | adenosine-2',3'-cyclic monophosphate | 0.0094 | 0.008 | 0.3182 | NS | **↓** | **↓** | NS | NS | NS |
|  | Purine metabolism, (hypo)xanthine/inosine containing | inosine | 0.0094 | 0.0019 | 0.5454 | **↓** | **↓** | NS | NS | NS | NS |
|  | Pyrimidine metabolism, orotate containing | orotate | 0.0033 | 0.0008 | 0.1179 | **↑** | **↑** | NS | **↑** | NS | NS |
|  | Pyrimidine metabolism, thymine containing | 3-aminoisobutyrate | 0.0033 | <0.0001 | 0.2326 | **↑** | **↑** | **↑** | NS | NS | NS |
|  | Pyrimidine metabolism, uracil containing | 5,6-dihydrouridine | 0.0018 | 0.0077 | 0.8729 | **↓** | **↓** | NS | NS | NS | NS |
|  |  | beta-alanine | 0.0033 | <0.0001 | 0.0561 | **↑** | **↑** | NS | **↑** | NS | NS |
| Xenobiotics | Food component/plant | 2,3-dihydroxyisovalerate | 0.0000 | 0.0952 | 0.0002 | NS | NS | **↑** | **↑** | **↑** | NS |
|  |  | 2-piperidinone | 0.0072 | 0.0002 | 0.16 | **↑** | **↑** | NS | NS | NS | NS |
|  |  | 3-formylindole | 0.0095 | <0.0001 | 0.0434 | NS | **↑** | NS | NS | **↑** | NS |
|  |  | mannonate | 0.0044 | 0.0815 | 0.0049 | NS | NS | NS | **↑** | NS | NS |

**Supplementary Table 5.** **Fecal metabolites with a significant group x condition interaction in the linear mixed-effects models (FDR-adjusted *p*-value<0.01) (*N*=63), with their respective super pathways and pathways**. The table compares the change (increase or decrease) of the fecal metabolites with a significant group x condition interaction in the linear mixed-effects models (LMMs) among the long-term alcohol drinking group (L), the short-term alcohol drinking group (S), and the control group (C), in the drinking (D) and abstinence (A) conditions, respectively. A green arrow (**↑**) indicates a significantly higher metabolite level in a group of baboons compared to another group. A red arrow (**↓**) indicates a significantly lower metabolite level in a group of baboons compared to another group. Non-significant (NS) differences are also shown. The table also displays FDR-adjusted *p*-values for the main effect of group in condition D, the main effect of group in condition A, and the interaction effect between group and condition.
