# Supplementary figures and images for: Gut microbiome and metabolome in a non-human primate model of chronic excessive alcohol drinking
Source: Transl Psychiatry. 2021 Dec 1;11:609. doi: 10.1038/s41398-021-01728-6 (PMC8636625; doi:10.1038/s41398-021-01728-6)

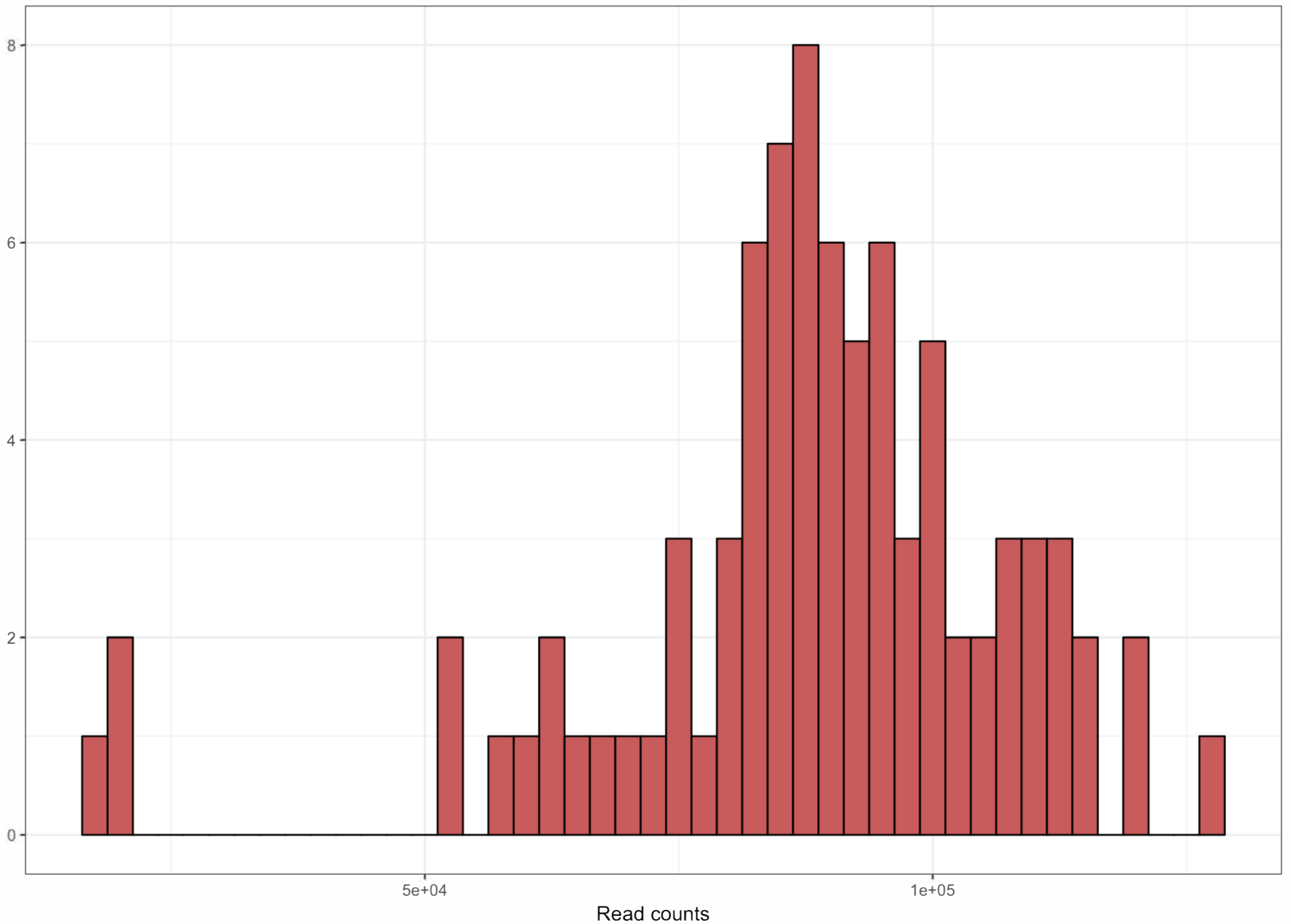

Supplement: Supplementary file 2 — Supplementary Fig. 1 [file 41398_2021_1728_MOESM2_ESM.pdf]

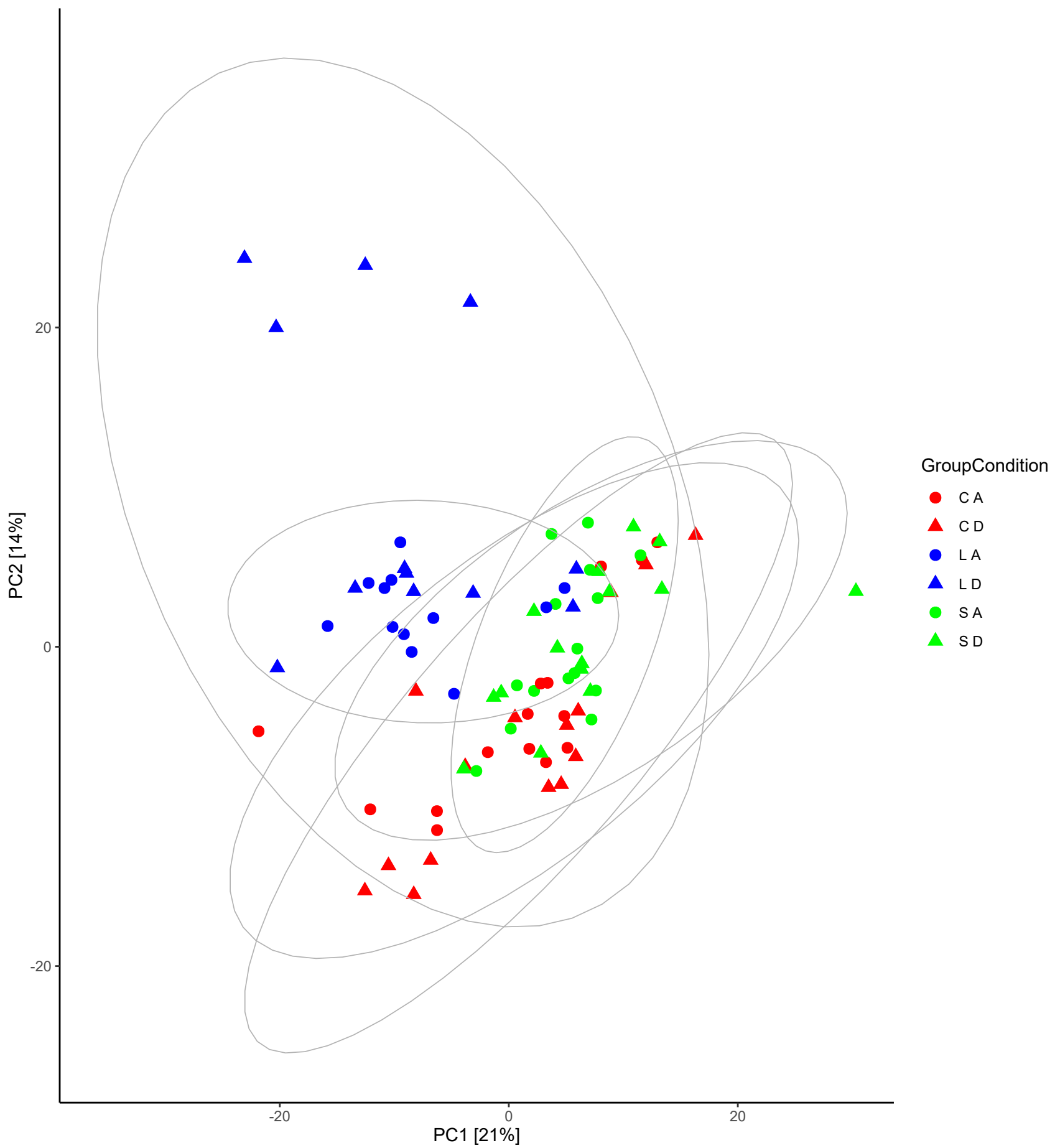

Supplement: Supplementary file 3 — Supplementary Fig. 2 [file 41398_2021_1728_MOESM3_ESM.pdf]
